# Ethnic variations in compulsory detention under the Mental Health Act: a systematic review and meta-analysis of international data

**DOI:** 10.1016/S2215-0366(19)30027-6

**Published:** 2019-04

**Authors:** Phoebe Barnett, Euan Mackay, Hannah Matthews, Rebecca Gate, Helen Greenwood, Kevin Ariyo, Kamaldeep Bhui, Kristoffer Halvorsrud, Stephen Pilling, Shubulade Smith

**Affiliations:** a Department of Clinical Educational and Health Psychology, Centre for Outcomes Research and Effectiveness, University College London, London, UK; b National Collaborating Centre for Mental Health, Royal College of Psychiatrists, London, UK; c Centre for Psychiatry, Wolfson Institute of Preventive Medicine, Barts and the London School of Medicine and Dentistry, Queen Mary University of London, London, UK; d Camden and Islington NHS Foundation Trust, London, UK; e Department of Forensic and Neurodevelopmental Science, Institute of Psychiatry, Psychology and Neuroscience, Kings College London, London, UK; f Bethlem Royal Hospital, South London and Maudsley NHS Foundation Trust, Beckenham, UK

## Abstract

**Background:**

Evidence suggests that black, Asian and minority ethnic (BAME) groups have an increased risk of involuntary psychiatric care. However, to our knowledge, there is no published meta-analysis that brings together both international and UK literature and allows for comparison of the two. This study examined compulsory detention in BAME and migrant groups in the UK and internationally, and aimed to expand upon existing systematic reviews and meta-analyses of the rates of detention for BAME populations.

**Methods:**

For this systematic review and meta-analysis, we searched five databases (PsychINFO, MEDLINE, Cochrane Controlled Register of Trials, Embase, and CINAHL) for quantitative studies comparing involuntary admission, readmission, and inpatient bed days between BAME or migrant groups and majority or native groups, published between inception and Dec 3, 2018. We extracted data on study characteristics, patient-level data on diagnosis, age, sex, ethnicity, marital status, and occupational status, and our outcomes of interest (involuntary admission to hospital, readmission to hospital, and inpatient bed days) for meta-analysis. We used a random-effects model to compare disparate outcome measures. We assessed explanations offered for the differences between minority and majority groups for the strength of the evidence supporting them. This study is prospectively registered with PROSPERO, number CRD42017078137.

**Findings:**

Our search identified 9511 studies for title and abstract screening, from which we identified 296 potentially relevant full-text articles. Of these, 67 met the inclusion criteria and were reviewed in depth. We added four studies after reference and citation searches, meaning 71 studies in total were included. 1 953 135 participants were included in the studies. Black Caribbean patients were significantly more likely to be compulsorily admitted to hospital compared with those in white ethnic groups (odds ratio 2·53, 95% CI 2·03–3·16, p<0·0001). Black African patients also had significantly increased odds of being compulsorily admitted to hospital compared with white ethnic groups (2·27, 1·62–3·19, p<0·0001), as did, to a lesser extent, south Asian patients (1·33, 1·07–1·65, p=0·0091). Black Caribbean patients were also significantly more likely to be readmitted to hospital compared with white ethnic groups (2·30, 1·22–4·34, p=0·0102). Migrant groups were significantly more likely to be compulsorily admitted to hospital compared with native groups (1·50, 1·21–1·87, p=0·0003). The most common explanations for the increased risk of detainment in BAME populations included increased prevalence of psychosis, increased perceived risk of violence, increased police contact, absence of or mistrust of general practitioners, and ethnic disadvantages.

**Interpretation:**

BAME and migrant groups are at a greater risk of psychiatric detention than are majority groups, although there is variation across ethnic groups. Attempts to explain increased detention in ethnic groups should avoid amalgamation and instead carry out culturally-specific, hypothesis-driven studies to examine the numerous contributors to varying rates of detention.

**Funding:**

University College London Hospitals National Institute for Health Research (NIHR) Biomedical Research Centre, NIHR Biomedical Research Centre at South London and Maudsley NHS Foundation Trust, King's College London, and NIHR Collaboration for Leadership in Applied Health Research and Care North Thames at Bart's Health NHS Trust.

## Introduction

Ethnic minorities are subject to a disproportionate risk of involuntary psychiatric detention.^1^, ^2^, ^3^ Research has suggested that patients from black, Asian and minority ethnic (BAME) groups have more compulsory admissions to hospital,^4^, ^5^ longer inpatient stays,^6^ and more readmissions.^7^ However, other studies have reported no or weak associations between BAME populations and increased detention.^8^, ^9^ Furthermore, the risk of involuntary psychiatric detention is not consistently higher in all or specific ethnic groups,^8^, ^9^, ^10^ potentially undermining actions to reduce inequalities and inform policy. Although evidence from the UK suggests increased detention under the Mental Health Act (MHA) for black populations,^11^, ^12^ less is known about south Asian (including Bangladeshi, Indian, Pakistani, Sri Lankan, and Nepali people)^9^, ^10^, ^13^, ^14^ and migrant populations.^15^ Several explanations have been suggested for the observed disparities between minority and majority ethnic groups, such as increased prevalence of schizophrenia in some minority ethnic^4^, ^11^ and migrant^16^, ^17^ populations, insufficient patient awareness of mental health issues,^10^, ^18^ more frequent adverse experiences with mental health services,^19^ experience of racism or health-care provider discrimination,^20^, ^21^ and differing use of psychiatric services.^22^, ^23^ However, few of these explanations provide adequate supporting evidence.^10^ A meta-analysis^3^ highlighted the importance of ethnic specificity in study design, considering black Caribbean patients separately and in addition to black patients more generally. However, there has otherwise been little evidence to systematically update and pool knowledge of the over-representation of BAME populations in those detained under the MHA in England since its revision and, to our knowledge, no systematic review or meta-analysis has been done from an international perspective. This study aims to expand upon existing systematic reviews and meta-analyses^1^, ^3^, ^10^ on the rates of detention for specific BAME populations from an international perspective, and outline explanations for any disparities between populations. Novel considerations of migrant populations are also reported.

**Research in context**

**Evidence before this study**

A large body of evidence suggests that black, Asian, and minority ethnic (BAME) groups are at an increased risk of compulsory detention under the Mental Health Act in England and Wales. However, there is marked variation in the composition of ethnic groups, definitions of ethnic groups, sample sizes, and reported magnitude of the effect sizes. The literature has typically focused on a small number of countries (primarily the UK) and has often neglected migrant populations. We searched MEDLINE (between Jan 1, 1946, and Nov 27, 2017), and PsycINFO (between Jan 1, 1806, and Nov 13, 2017), for systematic reviews and meta-analyses published in English with the search terms “minority groups” or “ethnic groups” or “BME” or “BAME” or “immigrants” or “refugees” and “Mental Health Act” or “commitment” or “admission” and “psychosis” or “schizophrenia” or “psychotic disorders”. We found no meta-analyses covering both the international and UK literature.

**Added value of this study**

To our knowledge, this is the first systematic review and meta-analysis to review both international and UK-based studies of compulsory detention, and the first to consider compulsory detention in migrant populations. This systematic review benefits from separate consideration of different ethnic groups where possible, avoiding grouping of culturally diverse populations. UK-based and international research showed significantly increased compulsory detention in several different ethnic minority and migrant populations, although UK research showed a more pronounced result.

**Implications of all the available evidence**

Our findings support that compulsory detention and readmission in all BAME populations is significantly increased compared with majority groups, as is that of migrant populations compared with host nation populations. Detention rates vary across different BAME groups, with the highest rate seen in black Caribbean populations, and less marked, but still significantly increased rates in south Asian populations. Future research should aim to establish the causes of ethnic disparities in involuntary care and should avoid cultural stereotypes and assumptions. Amalgamation of ethnic groups should be discouraged to better inform policy and practice.

## Methods

### Search strategy and selection criteria

For this systematic review and meta-analysis, we included studies of samples in which two or more ethnic groups of any age were compared, and that compared the risk of compulsory inpatient psychiatric care in minority and majority ethnic groups. Included study outcomes were compulsory inpatient admission to hospital, compulsory inpatient readmission to hospital, and inpatient length of stay, and we considered only quantitative data.

We developed our search strategy in consultation with an information scientist with experience in mental health, with a combination of keyword and subject heading searches. We searched MEDLINE (between Jan 1, 1946, and Nov 27, 2017), PsycINFO (between Jan 1, 1806, and Nov 13, 2017), Embase (between Jan 1, 1974, and Nov 20, 2017), Cochrane Controlled Register of Trials (between inception and Nov 27, 2017), and CINAHL (between Jan 1, 1981, and Nov 30, 2017).

Although our search was not confined to countries in which black and other non-white ethnicities are minorities, all papers meeting inclusion criteria used either white or the dominant national group as their comparison group. Full search strategies are available in the appendix (pp 1–20).

We contacted study authors; however, none contacted replied. We did not assess grey literature sources. Articles were translated to English, but none of the translated articles ended up being included. We sought summary estimate data rather than individual patient-level data.

Two reviewers (EM and KA) independently screened all titles and abstracts identified and excluded studies that did not meet the inclusion criteria. Full articles were subsequently reviewed in duplicate and in cases of disagreement, consensus was achieved through the referral of a third senior reviewer (HM). An update search was done on Dec 4, 2018, to identify any additional papers published between Nov 1, 2017, and Dec 3, 2018. We supplemented the search strategy with a backward reference search of included studies and a forward citation search using Scopus. References for all included studies are available in the appendix (pp 44–46).

The main change to our methods following protocol registration was that we expanded our inclusion criteria to encompass international literature in addition to literature from England and Wales. This change was made to provide an international context for our study, to further our understanding, and to include migrant populations, who are focused on more frequently in the international literature. Additionally, we did post-hoc meta-regression analyses to explore potential associations between predictor variables and ethnicity and to account for heterogeneity.

### Data analysis

Four independent reviewers (PB, EM, HG, and KA) extracted the data and all extraction was reviewed for accuracy. An electronic Microsoft Excel-based form was used to record data extraction. We planned to exclude studies which reported data already included in our dataset, but we did not find any duplicates.

We calculated overall summary estimates (odds ratios [ORs]) and 95% CIs with a random-effects model using the R package metafor version 2.0.^24^ P<0·05 was considered to indicate a significant difference. We used ORs because most papers identified in our search either provided the number of events and sample sizes to calculate ORs, or gave statistics in the format of ORs when raw data were not provided.

Studies varied in their specificity of classification of ethnic groups. Unlike previous studies, we attempted to avoid aggregate comparisons. Where possible, we organised studies into the subgroups black Caribbean, black African, south Asian, and east Asian. We classified studies solely reporting data for black or black, other groups as black, unspecified. We constructed a further non-specific classification of BAME, unspecified to contain studies reporting a mixture of minority ethnicities, for example, non-white British. We also did an analysis of studies comparing migrant groups (those born outside the host country) with host populations. We included only unadjusted data in the main analysis.

We did four post-hoc meta-regressions with Comprehensive Meta-Analysis software (version 3) to explore possible causes of heterogeneity and to investigate differences between UK and international literature. With these meta-regressions, we examined possible predictors of the effect of ethnicity on compulsory admission and included mean age, proportion of women, publication year, and national context (England and Wales or internationally). We also did sensitivity analyses, including only studies rated as high quality or higher ethnic specificity for the primary outcome of compulsory admission.

Four reviewers (PB, EM, HG, and KA) quality assessed the included studies. First, we applied the 14-item quality assessment checklist devised by Kmet and colleagues^25^ to each study. Each study was assessed against the 14 items using a 3-point scale with a score of 2 representing fully met, 1 representing partially met, and 0 meaning a study did not meet the criterion. A total score was calculated by adding up the scores achieved for each item. If a criterion was not applicable, it was excluded from the score calculation, and therefore from the maximum total score that could be achieved. A summary score (total sum divided by the total possible sum) was then calculated, representing the methodological quality of each study. These scores were calculated as a linear score from 0 to 100 and divided into three categories: low (≤49), moderate (50–74), or high (≥75) quality studies. Second, we assessed the quality of each study in terms of ethnic specificity with an adapted version of Raine's^26^ review of gender differences within health care, which was developed by Bhui and colleagues^1^ and has been used in previous similar reviews.^10^ Quality rating scores were between 0 and 14 and were categorised as follows: 0–3 (low), 4–7 (medium), and 8–14 (high). For both scales, quality assessment was discussed until a consensus was obtained and disagreements were resolved through consultation with two senior reviewers (SP and HM).

We extracted data on study design, sample size, population type, country, diagnosis, age, sex, ethnicity, marital status, living status (living alone, with family, or with a significant other), education, occupation, the legal system or act (eg, whether it was the Mental Health Act, and if yes which version, or if a different country, which legal ruling the person was detained under, although this was rarely reported), and the previously mentioned study outcomes of interest (ie, involuntary admission to hospital, readmission to hospital, and inpatient bed days) and their associated statistical data. As in the study by Singh and colleagues,^10^ we extracted explanations for differences in psychiatric detention of BAME groups from included studies. We summarised these explanations and recorded any support from primary evidence (data from the paper itself). Unsupported explanations were those that were untested by the design of the study. We classified explanations into five domains adapted from Singh and colleagues:^10^ patient-related, illness-related, service-related, culture-related, and service-patient interface. If studies were previously summarised by Singh and colleagues,^10^ those explanations were retained. We reported explanations only when primary evidence for an association was identified.

We assessed the degree of publication bias by visual examination of funnel plots.^27^

We calculated heterogeneity between studies with the *I*^2^ statistic. A value of 0% indicated no observed heterogeneity and 25%, 50%, or 75% tentatively signified low, moderate, or high heterogeneity between studies, respectively.^28^

This study follows the PRISMA guidelines^29^ and is prospectively registered with PROSPERO, number CRD42017078137.

### Role of the funding source

The funders of the study had no role in study design, data collection, data analysis, data interpretation, or writing of the report. The corresponding author had full access to all the data in the study and had final responsibility for the decision to submit for publication.

## Results

Our search identified 9511 studies for title and abstract screening, from which 296 potentially relevant full-text articles were identified (figure). Of these, 67 studies met inclusion criteria and were reviewed in depth. We also included an additional four studies after reference and citation searches. An updated search done on Dec 4, 2018, for studies published between Nov 1, 2017, and Dec 3, 2018, found no additional studies that met inclusion criteria. 1 953 135 participants were included in the studies we reviewed.

**Figure:**
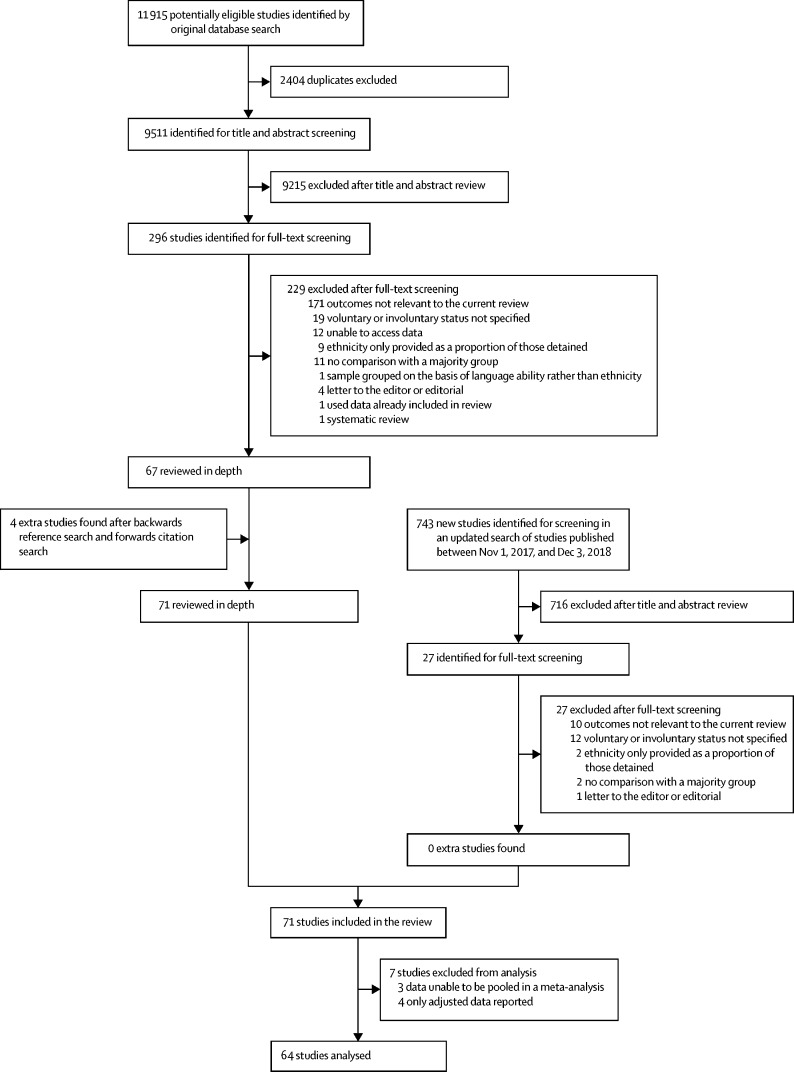
Study selection

Study characteristics are summarised in table 1. The 71 studies included in our review reported compulsory admission (63 studies), compulsory readmission (11 studies), and inpatient bed days (two studies). Two studies reported rate ratios^7^, ^47^ and one reported a risk ratio,^46^ which could not be pooled and included in our meta-analysis. Four studies reported adjusted data only so were also excluded from our main analysis.^22^, ^35^, ^82^, ^90^ Most studies reported routine data from specific hospitals or districts, although some compared population rates of admission. Studies were from high-income countries, and predominantly the UK (49 studies). Other countries represented were Canada (two studies), Italy (three studies), Ireland (two studies), the Netherlands (four studies), USA (five studies), Norway (one study), Switzerland (two studies), Denmark (one study), Spain (one study), and New Zealand (one study). We found high variability in study quality and scores awarded on the ethnicity checklist,^11^, ^26^ with scores ranging from 2 to 12. The main areas of bias centred on insufficient consideration of confounding variables. We examined funnel plots to investigate publication bias and found that studies were evenly distributed around the SE (appendix), suggesting that publication bias did not significantly affect our results. However, we observed high heterogeneity for all outcomes (table 2).

**Table 1:** Characteristics of included studies

	**Study type**	**Sample size, n**	**Outcomes**	**Population**	**Country**	**Mean age (range), years**	**Sex, % female**	**Ethnicity**	**Study quality**	**Ethnicity checklist**
Afuwape et al^30^ (2006)	Cohort	213	Compulsory admission	Community	England	37·5 (NR)	16·4	White: 55%; black Caribbean: 8%; black African: 10%; black British: 26%	High	Medium
Agius et al^31^ (2008)	Cohort	61	Compulsory admission	Community	England	NR	24·2	Caucasian: 43%; African Caribbean: 16%; south Asian: 41%	Low	Low
Ajnakina et al^32^ (2017)	Cohort	245	Compulsory admission	Clinic	England	NR (18–65)	44·0	White British: 38%; black African: 34·7%; black Caribbean: 27·3%	High	Medium
Ali et al^13^ (2007)	Cohort	294 387	Compulsory admission	Clinic	England	NR (18–65)	NR	Caucasian: 81% (of those detained); Asian: 19% (of those detained)	Moderate	Medium
Archie et al^33^ (2010)	Cross-sectional	200	Compulsory admission	Clinic	Canada	NR (16–50)	22·0	White: 60·2%; black: 15·4%; Asian: 12·5%	High	Medium
Balducci et al^34^ (2017)	Case-control	848	Compulsory admission	Hospital	Italy	41·6 (NR)	NR	Foreign nationality: 15·6%; native: 84·4%	Moderate	Medium
Bansal et al^35^ (2014)	Cohort	NR	Compulsory admission	Clinic and community	Scotland	46·3 (NR)	NR	NR	High	Medium
Bebbington et al^4^ (1994)	Cross-sectional	376	Compulsory admission	Hospital	England	NR	52·9	White: 79·5%; black Caribbean: 20·5% (of those compulsorily detained)	Moderate	Medium
Bhui et al^11^ (1998)	Cross-sectional	277	Compulsory admission	Prison	England	NR	0	White: 70%; black Caribbean:11%; black African: 6%; black British: 5%; Asian or other: 7%	High	High
Borschmann et al^36^ (2010)	Case-control	887	Compulsory admission	Community	England	37·1 (NR)	NR	White: 71·6%; black: 17·2%; Asian: 6·7%; mixed: 1·8%; Chinese: 1·7%	Low	Medium
Bowers et al^37^ (2009)	Cross-sectional	NR	Compulsory admission	Hospital	England	NR	50·9	White: 73·13%; Irish: 2·3%; Caribbean: 6·5%; African: 5·3%; south Asian: 6·4%; other: 6·4%	Moderate	High
Burnett et al^38^ (1999)	Cohort	909	Compulsory admission, readmission	Hospital and community	England	NR (17–62)	35·0	White: 38%; African Caribbean: 38%; Asian: 24%	Moderate	Medium
Callan^39^ (1996)	Cohort	144	Compulsory admission	Hospital	England	NR	39·5	White British: 51%; African Caribbean: 52%	Moderate	Medium
Coid et al^40^ (2000)	Case-control	3155	Compulsory admission	Hospital	England and Wales	31·4 (NR)	13·7	White: 74%; black: 21%; Asian: 3%; other: 2%	High	Medium
Cole et al^41^ (1995)	Case-control	93	Compulsory admission	Hospital	England	29·0 (17–53)	46·2	White: 42%; black Caribbean: 20%; black African: 15%; black other: 5%; Indian: 5%; Pakistani: 3%; other Asian: 3%	Moderate	Medium
Commander et al^42^ (1999)	Cohort	240	Compulsory admission	Hospital	England	NR (16–60)	50·4	White: 33%; black: 33%; Asian: 33%	Moderate	Medium
Cope and Ndegwa^43^ (1990)	Case-control	115	Compulsory admission, readmission	Hospital	England	32·0 (18–70)	10·4	White: 56·5%; African Caribbean: 38·3%; Asian: 5·2% (excluded)	Moderate	Medium
Corrigal and Bhugra^12^ (2013)	Case note review	435	Compulsory admission	Hospital	England	16·3 (12–17)	53·0	White: 32%; black: 49%; Asian: 3%; other: 16%	High	Medium
Crowley and Simmons^44^ (1992)	Case note review	152	Compulsory admission	Community and hospital	England	NR	46·7	White: 49·3%; African Caribbean: 50·7%	Moderate	Medium
Curley et al^45^ (2016)	Case-control	1099	Compulsory admission	Hospital	Ireland	40·2 (16·4–81·9)	52·9	Irish: 86·2%; other European: 6·82%; Asian: 1·36%; African: 4·73%; American: 0·09%	Moderate	Low
Davies et al^46^ (1996)	Cohort	439	Compulsory admission	Community and hospital	England	42·7 (NR)	52·6	White: 61·5%; Caribbean: 27·8%; black African: 7·0%; other: 3·6%	Moderate	Medium
de Wit et al^47^ (2012)	Case-control	2646	Compulsory admission	Hospital	Netherlands	NR	40·9	Dutch: 1289; Surinamese: 404; Antillean: 74; Moroccan: 169; Turkish: 96; other western: 384; other non-western: 230	Moderate	High
Dunn and Fahy^48^ (1990)	Case note review	268	Readmission	Hospital	England	33·2 (18–85)	37·9	White: 61%; black: 33%; Asian: 3% (excluded)	Low	Low
Fassaert et al^49^ (2016)	Case-control	30 655	Compulsory admission	Clinic	Netherlands	36·5 (NR)	38·1	Dutch natives: 58·8%; Antillean: 1·4%; Surinamese: 4·3%; Moroccan: 4%; Turkish: 2·3%; other non-western: 6·5%; other western: 6%; ethnicity unknown: 17·7%	High	High
Gajwani et al^5^ (2016)	Cohort	863	Compulsory admission	Community and hospital	England	NR	NR	White: 51·1%; Asian Pakistani: 14·9%; African Caribbean: 14%; black African: 7%	Moderate	Medium
Goater et al^50^ (1999)	Cohort	93	Compulsory admission	Community and hospital	England	NR	NR	White: 41·9%; black: 40·9%; Asian: 11·8%; other: 5·4%	Moderate	Medium
Gray Houston et al^51^ (2001)	Cohort	487	Compulsory admission	Hospital	USA	35·8 (18–65)	52·0	Euro-American: 53%; African-American: 34%; Hispanic American: 13%	Low	Low
Hamilton et al^52^ (2015)	Cohort	5183	Compulsory admission	Hospital	USA	35·7 (NR)	38·1	African American: 51·6%; non-Hispanic white: 48·4%	Moderate	Medium
Harrison et al^53^ (1984)	Case note review	203	Readmission	Hospital	England	NR	NR	White: 86·7%; West Indian: 13·3%	Low	Low
Ineichen et al^54^ (1984)	Cohort	264	Compulsory admission	Hospital	England	NR	60·3	NR	Low	Medium
Iverson and Morgan^55^ (2003)	Case-control	3053	Compulsory admission	Hospital	Norway	41·1 (NR)	48·7	Immigrants: 35·3%; asylum seekers: 14·7%; Norwegian: 50%	Moderate	Medium
Dannerbeck Janku and Yan^8^ (2009)	Case-control	379	Compulsory admission	Prison	USA	NR	NR	Caucasian: 48%; African American: 52%	Moderate	Medium
Johnson et al^56^ (1998)	Cohort	286	Compulsory admission	Community	England	42·1 (NR)	52·0	White: 63%; black Caribbean: 26%; black African: 6%; other: 5%	Moderate	High
Kelly et al^57^ (2015)	Cohort	518	Compulsory admission	Hospital	Ireland	40·1 (16–80)	49·6	Irish: 84%; other European: 6·6%; Asian: 2·3%; African: 5·6%; American: 0·8%; Australian: 0·8%	Low	Low
Kilbourne et al^58^ (2005)	Cross-sectional	330	Compulsory admission	Hospital	USA	46·6 (NR)	10·0	White: 76%; American Indian or Alaska native: 4%; Asian or Pacific islander: 5%; black: 14%; Hispanic: 5%; other: 5%	Moderate	Medium
Koffman et al^59^ (1997)	Cross-sectional	3978	Compulsory admission	Hospital	England	NR	NR	White: 75%; black:16%; Asian: 4%	Moderate	Medium
Lawlor et al^60^ (2012)	Case-control	287	Compulsory admission	Hospital	England	40·1 (18–69)	100	White British: 50·9%; white other: 15·7%; black Caribbean: 9·1%; black African: 14·3%; black other: 10·1%	High	High
Law-Min et al^61^ (2003)	Case-control	189	Compulsory admission, readmission	Hospital	England	40·0 (NR)	40·0	White: 66%; African Caribbean: 16%; Asian: 15%; other: 3%	Low	Low
Lay et al^22^ (2005)	Case-control	23 377	Compulsory admission	Hospital	Switzerland	37·3 (18–65)	47·0	Switzerland: 80·6%; southern Europe: 5·3%; west or northern Europe: 3%; former Yugoslavia: 4·3%; Turkey: 1·9%; eastern Europe: 0·8%; other: 4·2%	High	Medium
Lay et al^62^ (2011)	Cross-sectional	9698	Compulsory admission	Hospital	Switzerland	40·4 (18–70)	51·0	Switzerland: 78·3%; foreign national: 21·7%	High	High
Lindsey et al^63^ (1989)	Cross-sectional	227	Inpatient bed days, readmission	Hospital	USA	35·3 (18–65)	45·8	White: 50·7%; black: 49·3%	Moderate	Medium
Lloyd and Moodley^64^ (1992)	Cross-sectional	138	Compulsory admission	Hospital	England	NR	NR	White: 73·2%; black: 26·8%	Moderate	Medium
Mann et al^65^ (2014)	Cohort	674	Compulsory admission	Hospital	England	24·0 (18–35)	35·0	White British: 23·4%; white other: 13·8%; mixed: 5·3%; south Asian: 5·5%; other Asian: 4·3%; black British: 8·2%; black Caribbean: 11·6%; black African: 27·9%	High	Medium
McGovern et al^66^ (1994)	Cohort	75	Readmission	Hospital	England	22·2 (16–29)	30·6	White: 44%; black: 66%	Moderate	Medium
McKenzie et al^67^ (1995)	Cohort	113	Readmission	Hospital	England	NR (16–60)	30·9	White: 53·1%; African Caribbean: 46·9%	Moderate	High
Mohan at el^6^ (2006)	Cohort	140	Compulsory admission, inpatient bed days	Community	England	41·8 (NR)	43·8	White: 65·7%; African Caribbean: 34·3%	Moderate	Medium
Moodley and Perkins^68^ (1991)	Cross-sectional	52	Compulsory admission	Hospital	England	39·0 (18–64)	63·5	White: 48%; African Caribbean: 42%	Low	Medium
Morgan et al^69^ (2005)	Cohort	462	Compulsory admission	Hospital	England	NR (16–65)	42·2	White British: 51·3%; African Caribbean: 27·7%; black African: 13·9%; other white: 7·1%	Moderate	Medium
Mulder et al^23^ (2006)	Cohort	720	Compulsory admission	Community	Netherlands	37·0 (NR)	45·4	Dutch natives: 67%; immigrants: 33%	Moderate	Medium
Norredam et al^70^ (2010)	Cohort	312 300	Compulsory admission	Hospital	Denmark	30·0 (NR)	54·80	Refugees: 9·3%; immigrants: 10·7%; Danish born: 80%	Moderate	Medium
Oluwatayo and Gaterl^71^ (2004)	Case note review	200	Readmission	Hospital	England	33·0 (16–65)	41·0	White British: 50%; African Caribbean: 50%	Moderate	Medium
Owens et al^72^ (1991)	Case-control	275	Compulsory admission	Hospital	England	NR	49·8	White: 56·4%; African Caribbean: 43·6%	Moderate	Low
Parkman et al^73^ (1997)	Case note review	202	Compulsory admission	Community and hospital	England	43·2 (NR)	46·9	White: 73%; black Caribbean: 27%	Moderate	High
Perez-Rodriguez et al^74^ (2006)	Cohort	1015	Compulsory admission	Hospital	Spain	NR	NR	NR	Moderate	Medium
Priebe et al^7^ (2009)	Case-control	778	Readmission	Hospital	England	37·1 (18–65)	38·0	White: 73%; black: 18%; Asian: 6%; other: 3%	High	Medium
Reeves et al^75^ (2002)	Cohort	44	Compulsory admission	Hospital	England	NR	75·9	British born: 50%; Caribbean born: 50%	Moderate	Medium
Rotenberg et al^76^ (2017)	Cohort	765	Compulsory admission	Hospital	Canada	38·0 (NR)	36·7	East Asian: 17·9%; south Asian: 11·9%; black African: 12·9%; black Caribbean: 18·4%; white North American: 19·6%; white European: 19·2%	High	High
Selten and Sijben^77^ (1994)	Cohort	813	Compulsory admission	Hospital	Netherlands	NR (15–34)	0	Native Dutch: 83·4%; Surinamese: 2·6%; Antillean: 1·0%; Turkish: 0·5%; Moroccan: 1·7%	Low	Low
Singh et al^78^ (1998)	Cross-sectional	417	Compulsory admission	Hospital	England	NR (16–NR)	49·2	White European: 81·5%; black Caribbean: 7·3%; black African: 0·4%; Pakistani: 1·7%; Indian: 2·5%; Bangladeshi: 0·2%; Chinese: 0·2%; mixed ethnic origin: 2·8%	High	Medium
Singh et al^2^ (2014)	Cohort	4275	Compulsory admission	Hospital	England	NR	43·5	White: 61·6%; black: 19·3%; Asian: 10·4%; other: 8·6%	High	Medium
Singh et al^79^ (2015)	Cohort	123	Compulsory admission	Hospital	England	23·2 (14–37)	26·0	White: 36·6%; black: 28·4%; Asian: 35%	High	High
Sohler et al^80^ (2004)	Case-control	501	Compulsory admission	Hospital	England	NR	42·80	Black: 16·2%; white: 83·8%	Moderate	High
Spinogatti et al^81^ (2015)	Case-control	NR	Compulsory admission	NR	Italy	NR (17–NR)	NR	NR	Low	Medium
Takei et al^82^ (1998)	Cohort	88	Compulsory admission	Hospital	England	22·9 (18–44)	37·1	White: 60·5%; African Caribbean: 39·5%	Moderate	Medium
Tarsitani et al^83^ (2013)	Case-control	200	Compulsory admission	Hospital	Italy	35·9 (NR)	58·0	Natives: 50%; immigrants: 50%	Moderate	Medium
Thomas et al^84^ (1993)	Case-control	1534	Compulsory admission; readmission	NR	England	36·6 (16–NR)	47·9	UK: 82·5%; Asian: 4·9%; African Caribbean: 12·6%	Moderate	Medium
Thornicroft et al^85^ (1999)	Case note review	439	Compulsory admission	Hospital	England	42·7 (NR)	52·6	White: 57·9%; black Caribbean: 26·2%; black African: 6·6%; other: 3·4%	High	Medium
Tolmac and Hodes^86^ (2004)	Cross-sectional	113	Compulsory admission	Hospital	England	NR (13–17)	53·0	White: 60%; black: 19%; Asian: 10%; other: 11%	Moderate	Medium
Webber and Huxley^87^ (2004)	Case note review	300	Compulsory admission	Hospital	England	39·5 (NR)	48·6	White British: 62%; non-white British: 38%	Moderate	Medium
Weich et al^88^ (2017)	Cross-sectional	1 238 188	Compulsory admission	Hospital	England	NR	55·3	White: 80·2%; black: 3·4%; Asian: 4%; mixed: 1%; other: 2%	High	High
Wheeler et al^89^ (2005)	Case note review	932	Compulsory admission	Hospital	New Zealand	NR (16–68)	44	European: 60·3%; New Zealand Maori: 23·4%; Pacific nations: 10·7%; Asian: 4·3%; other 1·3%	Moderate	Medium

NR=not reported.

**Table 2:** Association of involuntary psychiatric care with ethnicity

	**Number of studies**	**Odds ratio (95% CI)**	**p value**	**I^2^**
**Compulsory inpatient admission**				
Black African	10	2·27 (1·62–3·19)	<0·0001	71·11%
Black Caribbean	25	2·53 (2·03–3·16)	<0·0001	70·69%
Black, unspecified	20	2·00 (1·28–3·11)	0·0022	98·08%
South Asian	20	1·33 (1·07–1·65)	0·0091	83·38%
East Asian	3	2·17 (1·47–3·22)	0·0001	8·88%
Other minority ethnicities	13	1·66 (1·29–2·14)	<0·0001	81·14%
Migrants	12	1·50 (1·21–1·87)	0·0003	87·15%
**Compulsory inpatient readmission**				
Black Caribbean	7	2·30 (1·22–4·34)	0·0102	81·95%
Black, unspecified	4	1·30 (0·69–2·44)	0·4118	66·87%
South Asian	2	2·34 (0·61–8·99)	0·2161	89·57%
**Compulsory inpatient bed days**				
Black, combined*	2	0·88 (0·18–4·19)	0·8687	83·61%

All groups were compared with white populations except migrants, who were compared with host nation populations.

* For inpatient bed days, black, combined comprises black and African Caribbean patients.

Black ethnic groups were significantly more likely to be compulsorily admitted to hospital compared with white ethnic groups (black, unspecified OR 2·00, 95% CI 1·28–3·11, p=0·0022; black Caribbean 2·53, 2·03–3·16, p<0·0001; black African 2·27, 1·62–3·19, p<0·0001; table 2). Black Caribbean patients were also significantly more likely to be readmitted to hospital compared with white ethnic groups (2·30, 1·22–4·34, p=0·0102). We found no significant association between ethnicity and inpatient bed days (0·88, 0·18–4·19, p=0·8687), although this comparison included only two studies (table 2). People from Asian ethnic groups were significantly more likely to be compulsorily admitted to hospital compared with people from white ethnic groups (south Asian 1·33, 1·07–1·65, p=0·0091; east Asian 2·17, 1·47–3·22, p=0·0001). Only two studies reported compulsory inpatient readmission in south Asian patients and the results of these were not significant. Other minority ethnicities were significantly more likely to be compulsorily admitted to hospital compared with majority groups (1·66, 1·29–2·14, p<0·0001), as were migrant populations compared with host nation populations (1·50, 1·21–1·87, p=0·0003). Forest plots for our analyses are provided in the appendix (pp 25–30).

Study location was a significant predictor of compulsory admission in black, unspecified groups, such that UK-based studies reported significantly increased odds of compulsory admission in black ethnic groups compared with international studies (table 3). The proportion of women in the sample was also a significant predictor of compulsory admission to hospital in black, unspecified, black Caribbean, and south Asian groups. This association remained significant when adjusted for age in black, unspecified and black Caribbean groups, but was no longer significant in south Asian groups. Publication date was a significant predictor of compulsory admission to hospital only in black Caribbean groups, and mean age was not a significant predictor of compulsory admission (table 3). Scatter plots of these data are provided in the appendix (p 31–35).

**Table 3:** Predictors of involuntary psychiatric admission by ethnicity

	**Number of studies**	**R^2^**	**p value**	**Coefficient (95% CI)**
**Publication date**				
Black African	10	0%	0·6157	−0·0126 (−0·0620 to 0·0367)
Black Caribbean	25	42%	0·0006	−0·0361 (−0·0567 to −0·0156)
Black, unspecified	20	7%	0·9626	0·0013 (−0·0543 to 0·0570)
South Asian	19	0%	0·6401	−0·0098 (−0·0403 to 0·0206)
Migrants	12	0%	0·4400	0·0146 (−0·0225 to 0·0517)
**Proportion of women**				
Black Caribbean	24	59%	<0·0001	0·0247 (0·0135 to 0·0359)
Black, unspecified	15	64%	0·0344	0·0191 (0·0014 to 0·0367)
South Asian	15	30%	0·0345	0·0178 (0·0003 to 0·0354)
**Mean age**				
Black Caribbean	12	25%	0·0646	0·0452 (−0·0019 to 0·0924)
**International study location**				
Black, unspecified	20	31%	0·0434	−0·8976 (−1·7685 to −0·0267)

All groups were compared with white populations except migrants, who were compared with host nation populations.

We did secondary analyses on compulsory admission data to examine the effect of study quality on results (table 4). When including only studies scoring highly on the ethnicity checklist, results remained significant. When including only studies rated highly with the Kmet quality assessment scale, results remained significant in all black ethnic groups, but became non-significant in south Asian groups. Only six studies scored highly on both quality assessment scales, making these analyses difficult to draw conclusions from.

**Table 4:** Association of involuntary inpatient admission and ethnicity, restricted to high-quality studies

	**Number of studies**	**Odds ratio (95% CI)**	**p value**	**I^2^**
**Black African**				
All studies	10	2·27 (1·62–3·19)	<0·0001	71·11%
Kmet study quality	6	2·63 (1·80–3·83)	<0·0001	44·85%
Ethnicity checklist	5	2·49 (1·62–3·82)	<0·0001	56·89%
Both	3	2·40 (0·84–6·89)	0·1038	77·39%
**Black Caribbean**				
All studies	25	2·53 (2·03–3·16)	<0·0001	70·69%
Kmet study quality	7	2·15 (1·48–3·13)	0·0001	56·53%
Ethnicity checklist	6	2·45 (1·81–3·32)	<0·0001	32·86%
Both	3	2·03 (0·86–4·77)	0·1045	65·92%
**Black, unspecified**				
All studies	20	2·00 (1·28–3·11)	0·0022	98·08%
Kmet study quality	9	2·25 (1·15–4·37)	0·0172	98·01%
Ethnicity checklist	5	3·31 (1·72–6·38)	0·0003	84·41%
Both	4	4·35 (4·22–4·49)	<0·0001	0
**South Asian**				
All studies	20	1·33 (1·07–1·65)	0·0091	83·38%
Kmet study quality	8	1·07 (0·71–1·61)	0·7543	89·89%
Ethnicity checklist	4	1·89 (1·82–1·97)	<0·0001	0
Both	3	1·90 (1·83–1·98)	<0·0001	0

Other groups had insufficient numbers of high quality or high ethnicity checklist scoring studies for analysis.

We did a sensitivity analysis to investigate if studies excluded for reporting only adjusted data^22^, ^35^, ^82^, ^90^ could have significantly affected results (appendix p 36). We included three studies in the sensitivity analysis because they adjusted for demographic variables (age and sex) only.^22^, ^82^, ^90^ We observed only marginal differences, with no consequent difference in interpretation. One study^35^ adjusted for additional variables (car ownership and housing tenure) in addition to age and sex and reported only risk ratios; we excluded this study from the sensitivity analysis.

We extracted explanations for disparities in psychiatric detentions from all papers included in the review (appendix pp 37–43). Of the 71 studies, 12 offered no explanation for differences in psychiatric detention of BAME groups, 21 solely offered explanations unsupported by primary evidence, and 38 offered at least one explanation supported by primary evidence (appendix pp 37–43). 24 classifications of explanations emerged over five domains (appendix p 37), of which ten were unsupported by any of the included literature, seven had a mix of supporting and contradictory primary evidence, and seven were supported by primary evidence. The most common explanations with supporting evidence included increased prevalence of psychosis, increased perceived risk of violence, increased police contact, absence of or mistrust of general practitioners, and ethnic disadvantages. By contrast, frequently mentioned unsupported explanations for disparities in detention included higher comorbid drug use in BAME groups, language barriers, poorer detection of mental illness, and greater stigma than in majority groups.

## Discussion

This review expands on previous research on the use of involuntary psychiatric detention in ethnic minority communities, through examination of both UK and international data. Black ethnic groups (black Caribbean, black African, and black, unspecified) were more likely to be involuntarily admitted to hospital compared with those of white ethnicity. Black Caribbean individuals also had an increased risk of readmission to hospital. South Asian groups had a significantly increased risk of involuntary admission, as did east Asian patients, although interpretation was restricted by small study numbers. Our observed associations between compulsory admission to hospital and ethnicity remained significant when restricted to studies we assessed as being of high ethnic specificity. Following restriction of analysis to studies with a high methodological quality rating, only the south Asian association became non-significant. This result could suggest that less methodologically sound studies drove the reported higher risk for detention in south Asian populations. By contrast, continued significance when examining only studies with high ethnic specificity (regardless of other methodological aspects) contradicts this result. We could draw the most confidence from studies of both high methodological quality and clarity and consistency of ethnic classification, but only seven studies met these criteria.

Both ethnically and culturally heterogeneous BAME, unspecified and migrant groups also had an increased risk of involuntary admission to hospital, which suggests that, although effect estimates were lower than some previous literature,^1^, ^10^ all minority populations in the countries studied, including migrants, are subject to increased risks of detention.

Publication date predicted an association between black Caribbean ethnicity and involuntary care, with more recent studies reporting lower effect estimates. This result might reflect more rigorous study designs in recent literature. UK-based studies also showed a higher risk of compulsory admission to hospital for black, unspecified groups compared with international literature. Despite our attempts to provide specific ethnic classifications, the high heterogeneity for all groups could also reflect this variation in the UK and internationally. However, this effect is difficult to disentangle because the international studies were few and typically lacked the specificity of ethnic classification more common to British studies, preventing further post-hoc examination across all groups. The proportion of women in the sample strengthened associations in black Caribbean, black, unspecified, and south Asian groups. However, whole sample proportions cannot adequately describe intersectional experiences of race and sex^91^ and future robust investigation is required. Data on readmission to hospital and length of stay were scarce, and socioeconomic and clinical moderators by ethnic group and involuntary status were infrequently reported, preventing meaningful investigation.

Of the 71 papers included in our systematic review, 34 (48%) offered no explanation for the variation in risk of detention among minority groups, or solely offered explanations without support from primary evidence. Untested explanations perpetuated in the literature largely dealt with lifestyle, cultural health beliefs, clinical characteristics, and demographic-bound assumptions of minority ethnic groups (eg, more drug use and greater community stigma of mental illness). Such untested hypotheses are of little use and are problematic when applied to aggregated and non-specific ethnic groupings, which often contain populations with varying lifestyles, health beliefs, culture, religion, and other demographic variables. Application of assumptions to combined groups, which fail to consider intersectionality in the perpetuation of risk,^92^, ^93^ precludes further inquiry into the range of risks to which these groups are subject. Likewise, explanations with supporting evidence, such as increased rates of psychoses in minority groups, require close examination. Psychosis alone is not a criterion for detention under the MHA, therefore the prevalence of psychoses in BAME communities is insufficient to explain ethnic inequalities in detention. The excess of detentions in BAME groups also applies to readmissions and not just those presenting with a first episode, so might be a function of the care experienced by those with established psychoses. Differences leading to mental illness trajectories that result in detention, such as perceived risk of violence, also warrant further investigation.^23^, ^64^

This study has several limitations. First, included studies examined psychiatric hospitalisation only. This investigation provides a good basis for examining inequalities in psychiatric care, but ethnic differences in other psychiatric contexts should also be examined through robust research. Additionally, the insufficient data on other aspects of care provided in most studies and the pooling of data in a meta-analysis cannot provide the necessary detail on the nature of differences in admissions, both for compulsory detention and treatment where the patient agrees to care. Coercive non-formal admissions can also happen, and perceived coercion can substantially affect a patient's experience of care.^94^ Furthermore, both civil and forensic commitment were combined in the analysis. This strategy allowed a broader inclusion of literature, but important differences between the two forms of compulsory hospital admission could have been missed. Similarly, important differences in legal systems in the different countries included in this systematic review should not be ignored. However, we believe that providing international data on ethnic disparity gives a clearer picture of the shortcomings of present research in tackling a global problem.

To our knowledge, this systematic review and meta-analysis is the most comprehensive to date on ethnicity and involuntary psychiatric hospitalisation, integrating international comparisons and psychiatric detention of migrants. However, a substantial portion of the literature presented lacked the methodological quality to allow us to draw mechanistic or causal inferences from it. The included studies provide restricted information on socioeconomic, cultural, and structural determinants of detention, and integration of data on detention with such factors is an important area for further research. Retention of untested explanations in the studies covered by this systematic review might serve to entrench narratives of racial determinism and contribute little to a fuller understanding of the range of inequalities faced by minority ethnic groups who come into contact with psychiatric services. Research should prioritise longitudinal study designs that can investigate clinical, socioeconomic, and demographic contributions, and avoid simple techniques to analyse complicated problems^95^—ethnicity is a complex construct that comprises multiple interacting variables. Research should also integrate qualitative assessment of service provider biases, group level stigma, or patient mistrust of health-care services to gain a thorough understanding of individual patient experience. Decision-making processes in psychiatric detention, which exclude patient and family input to risk management,^96^ should also be examined because their interaction with situational factors, such as available alternative treatment and under-resourced services,^97^ might reflect area deprivation experienced by BAME communities.

Panel **Lived experience commentary**

Steve Gilbert: “Reading the research presented in this systematic review makes it clear that although research has been done with the best of intentions and hopes for change, it has not been adequately rooted in the people it is trying to help. Attempts to highlight injustices have backfired and given ammunition to those people who are not willing to stop and think—we have left our research too open to interpretation. We need to be asking different questions when considering study design—that is, we should seek not just to say what the state of play is, but also why the state of play is how it is. Another key question is, who is doing the research? Research can and should be done by a range of people who have followed different trajectories. Relevant to ethnicity and compulsory detention, experience of the criminal justice system, ethnic discrimination, or low-income households will positively contribute to the conclusions drawn. Furthermore, the methodology of research to date has felt distant—it does not reflect real experience. Data are good and useful to contribute to a fuller understanding, but have we become entrenched in the data? It is wrong to assume that if data say something is true then it must be true?”

This meta-analysis showed that all minority populations studied were subject to an increased risk of involuntary psychiatric detention. We are no closer to understanding or effectively addressing these ethnic inequalities in psychiatric care. Only research committed to well-designed longitudinal studies and multisectoral, intersectional approaches will be able to untangle the causes of this health-care inequality.
