# Supplementary material for: Ethnic variations in compulsory detention under the Mental Health Act: a systematic review and meta-analysis of international data
Source: Lancet Psychiatry. 2019 Apr;6(4):305–17. doi: 10.1016/S2215-0366(19)30027-6 (PMC6494977; doi:10.1016/S2215-0366(19)30027-6)
Supplement: Supplementary appendix [file mmc1.pdf]

THE LANCET
Psychiatry

## Supplementary appendix

This appendix formed part of the original submission and has been peer reviewed. We post it as supplied by the authors.

Supplement to: Barnett P, Mackay E, Matthews H, et al. Ethnic variations in compulsory detention under the Mental Health Act: a systematic review and meta-analysis of international data. *Lancet Psychiatry* 2019; published online March 4. http://dx.doi.org/10.1016/S2215-0366(19)30027-6.

# Appendices

## Search Strategy: Databases and dates searched

| Name of database | Date of search from: | Date of search to: | Records retrieved | Notes |
|---|---|---|---|---|
| MEDLINE | 1946 | 11-11-17 | 2304 | |
| PsycINFO | 1806 | Week2-Nov-2017 | 4319 | (no study filter applied) |
| Embase | 1974 | Week47-2017 | 2515 | |
| CENTRAL & other CLib databases | All years | Issue 10, October 2017 | 1070 | |
| CINAHL | 1981 | 30-11-17 | 1671 | (no study filter applied) |
| Other (Ovid XSearch *(Review & MHA):Ti (additional records not retrieved by other searches)* | All available years | 30-11-17 | 36 | |
| | | Total | **11915** | |
| | | De-duplicated | 9511 | |

| Database(s): **Ovid MEDLINE(R) Epub Ahead of Print, In-Process & Other Non-Indexed Citations, Ovid MEDLINE(R) Daily and Ovid MEDLINE(R) 1946 to 27-Nov-2017** | | |
|---|---|---|
| **#** | **Searches** | **Results** |
| 1 | Minority Groups/ | 12974 |
| 2 | Minority Health/ | 664 |
| 3 | exp Population Groups/ | 285829 |
| 4 | exp Ethnic Groups/ | 147240 |
| 5 | (ethnic* or multi-ethnic* or multiethnic* or indig* or aborig* or native* or inuit* or eskimo* or kalaallit* or amerind* or romany or romani* or gypsies or gipsies).ti,ab,kf,hw. | 424738 |
| 6 | (african or africaan or caribbean* or afro-caribbean* or afrocaribbean* or asian* or bangladeshi* or chinese or indian* or pakistani* or somali*).ti,ab,kf,hw. | 572531 |
| 7 | (black or blacks).ti. or (black adj3 (patient? or men or women or adult? or adolesc* or teen* or young* or youth? or old* or elder*)).ti,ab,kf. | 49411 |
| 8 | (BME or BAME).ti,ab,kf. | 1348 |
| 9 | "Emigrants and Immigrants"/ | 10375 |
| 10 | Refugees/ | 8986 |
| 11 | Cultural diversity/ | 11130 |
| 12 | Cross Cultural Comparison/ | 26137 |
| 13 | exp Human Migration/ | 26128 |
| 14 | (refugee* or immigrant* or (asyl* adj1 seek*) or foreign* or ethnic* or minorit* or race or racial* or (multi adj cultural*) or multicultural* or (newly adj arrived) or ((family or families) adj2 reuni*) or resettlement).ti,ab,kf. | 364570 |
| 15 | (cultural or culture or race or racial or nonwhite? or non-white? or (non uk adj (born or national))).ti,ab,kf. | 770214 |
| 16 | (minority adj2 (health* or group?)).ti,ab,kf. | 7018 |
| 17 | (ethnology and utilization).fs. | 7036 |
| 18 | or/1-17 | 1801552 |
| 19 | Mental Health Act.ti,ab,kf. and statistics & numerical data.fs. | 185 |
| 20 | Commitment of Mentally Ill/ and ethnology.fs. | 121 |
| 21 | (mental health adj (act? or law? or legal* or legislat* or jurisprudence)).mp. | 1817 |
| 22 | Commitment of mentally ill/ | 7142 |
| 23 | (Mental Disorders/eh or exp Psychotic Disorders/eh) and (psychiatric morbidity.ti,ab,kf. or incidence/ or prevalence/) | 479 |
| 24 | or/21-23 | 8705 |

| | | |
|---|---|---|
| 25 | Emergency Services, Psychiatric/ | 2483 |
| 26 | Hospitals, psychiatric/ and (Crisis intervention/ or emergenc*.ti,kf,hw.) | 535 |
| 27 | (psychiatric adj3 (in-patients or inpatients or morbidity)).ti,ab,kf. | 10223 |
| 28 | *mental disorders/ | 125164 |
| 29 | exp "schizophrenia spectrum and other psychotic disorders"/ | 145117 |
| 30 | Forensic Psychiatry/ | 9307 |
| 31 | (((mental or psychiatr*) adj (health or disorder* or disease* or deficien* or illness* or problem*)) or psychotic or psychosis or psychoses or psychopath* or schizo* or SMI or bipolar or ((suicid* or self-harm) adj3 (risk or crisis or crises))).ti,ab,kf. | 464216 |
| 32 | ((psychiatr* or psychology or health or medicine) and (law or legal or legislat* or jurisprudence)).jw. | 40900 |
| 33 | or/25-32 | 587393 |
| 34 | ((commit* or admission? or admitt* or detain* or detention? or section* or hospitali#ation?) adj3 (coerc* or compuls* or forced or enforced or involuntary or in-voluntary or rate? or status)).ti,ab,kf. | 28627 |
| 35 | admission?.ab. /freq=2 | 65874 |
| 36 | acute admission?.ti,ab,kf. | 757 |
| 37 | ((police or policing or local authority polic*) and (admission? or referral?)).ti,ab,kf,hw. | 864 |
| 38 | ((prison* or custody or incarcerat*) adj3 (transfer* or transition* or referral?)).ti,ab,kf,hw. | 213 |
| 39 | legal status.ti,ab,kf. | 1522 |
| 40 | *hospitalization/ | 35808 |
| 41 | *Hospitals, Psychiatric/ut | 779 |
| 42 | Psychiatry Department, Hospital/ut | 0 |
| 43 | coercion/ | 4476 |
| 44 | (detention? or admission? or hospitali#ation? or institutionali#ed).ti,kf. | 42481 |
| 45 | ((treatment or restriction or court or compulsory) adj order?).ti,ab,kf. | 1273 |
| 46 | (hospitalization/ or patient admission/ or patient discharge/ or patient handoff/ or patient readmission/ or patient transfer/) and ethnology.fs. | 2117 |
| 47 | (Prevalence/ or Incidence/) and (mental health services/ or community mental health services/ or emergency services, psychiatric/) and utilization.fs. | 1011 |
| 48 | (pathway* adj3 (care or hospital* or psychiatr*)).ti,ab,kf. | 4872 |
| 49 | critical pathways/ | 6245 |
| 50 | pathway?.ti. | 177461 |
| 51 | "route? to care".ti,ab,kf. | 78 |

| | | |
|---|---|---|
| 52 | exp General Practice/ and diagnosis.fs. | 10952 |
| 53 | (continuity adj3 care).ti,ab,kf. and ((health services or community) and (followup or follow-up or interview* or questionnaire* or focus group*)).ti,ab,kf,hw. | 1173 |
| 54 | conditional* discharge?.ti,ab,kf. | 32 |
| 55 | revolving door?.ti,ab,kf. | 326 |
| 56 | (readmission? or re-admission? or recurrence?).ti,kf,hw. | 320825 |
| 57 | or/34-56 | 650917 |
| 58 | (18 and 24) or (18 and 33 and 57) | 3616 |
| 59 | randomized controlled trial.pt. | 505458 |
| 60 | (randomi#ed or randomi#ation or randomi#ing).ti,ab,kf. | 580948 |
| 61 | (RCT or "at random" or (random* adj3 (assign* or allocat* or control* or crossover or cross-over or design* or divide* or division or number))).ti,ab,kf. | 450978 |
| 62 | (control* and (placebo or waitlist* or wait* list* or ((treatment or care) adj2 usual) or no intervention* or non intervention* or non-intervention* or without any intervention*)).ti,ab,kf,hw. | 211050 |
| 63 | (control group? or (controlled adj (trial or study))).ti,ab,kf. | 538179 |
| 64 | ((single or double) adj blind*).ti,ab,kf,hw. | 232155 |
| 65 | comparative study.pt. | 1936005 |
| 66 | or/59-65 | 2955543 |
| 67 | 58 and 66 | 821 |
| 68 | Epidemiologic studies/ | 8123 |
| 69 | (epidemiologic* adj3 survey?).ti,ab,kf. | 10377 |
| 70 | exp case control studies/ | 974962 |
| 71 | exp cohort studies/ | 1866550 |
| 72 | (case control or case series or (case adj (records or reports))).ti,ab,kf. | 258883 |
| 73 | (cohort adj (study or studies)).ti,ab,kf. | 162615 |
| 74 | cohort analy$.ti,ab,kf. | 7226 |
| 75 | ((followup or follow-up) adj (study or studies)).ti,ab,kf,hw. | 661042 |
| 76 | ((followup or follow-up) adj5 (interview* or questionnaire* or focus group*)).ti,ab,kf. | 12677 |
| 77 | ((observational or comparative) adj (study or studies)).ti,ab,kf,hw. | 2088622 |
| 78 | (prospective or retrospective).ti,ab,kf. | 930060 |
| 79 | longitudinal.ti,ab,kf. | 217616 |
| 80 | cross sectional.ti,ab,kf,hw. | 396355 |

| | | |
|---|---|---|
| 81 | ((study or research) and (data or analys*)).ti,ab,kf. | 3057861 |
| 82 | ((qualitative or quantitative) adj (analysis or study or studies)).ti,ab,kf. | 103145 |
| 83 | or/68-82 | 6512693 |
| 84 | 58 and 83 | 2241 |
| 85 | meta-analysis.pt. or (meta-analys* or meta analys* or metaanalys* or meta synth* or meta-synth* or metasynth*).ti,ab,kf,hw. | 163036 |
| 86 | (systematic or structured or evidence or trials or studies).ti. and ((review or overview or look or examination or update* or summary).ti. or review.pt.) | 163508 |
| 87 | (0266-4623 or 1469-493X or 1366-5278 or 1530-440X or 2046-4053).is. | 17868 |
| 88 | meta-analysis.pt. or (meta-analys* or meta analys* or metaanalys* or meta synth* or meta-synth* or metasynth*).ti,ab,kf,hw. | 163036 |
| 89 | ((systematic or meta) adj2 (analys* or review)).ti,kf. or ((systematic* or quantitativ* or methodologic*) adj5 (review* or overview*)).ti,ab,kf,sh. or (quantitativ$ adj5 synthesis$).ti,ab,kf,hw. | 195064 |
| 90 | (integrative research review* or research integration).tw. or scoping review?.ti,kf. or (review.ti,kf,pt. and (trials as topic or studies as topic).hw.) or (evidence adj3 review*).ti,ab,kf. | 180005 |
| 91 | or/85-90 | 439179 |
| 92 | (22 or ((33 and (34 or 43 or 44 or 45)) or 58)) and 91 | 284 |
| 93 | 19 or 20 or 67 or 84 or 92 | 2720 |
| 94 | 18 and 33 and 36 | 15 |
| 95 | 93 or 94 | 2725 |
| 96 | remove duplicates from 95 | **2304** |

Final search string (#95):

(( Mental Health Act.ti,ab,kf. and statistics & numerical data.fs.) or (Commitment of Mentally Ill/ and ethnology.fs.) or (((((Minority Groups/) or (Minority Health/) or (exp Population Groups/) or (exp Ethnic Groups/) or ((ethnic* or multi-ethnic* or multiethnic* or indig* or aborig* or native* or inuit* or eskimo* or kalaallit* or amerind* or romany or romani* or gypsies or gipsies).ti,ab,kf,hw.) or ((african or africaan or caribbean* or afro-caribbean* or afrocaribbean* or asian* or bangladeshi* or chinese or indian* or pakistani* or somali*).ti,ab,kf,hw.) or ((black or blacks).ti. or (black adj3 (patient? or men or women or adult? or adolesc* or teen* or young* or youth? or old* or elder*)).ti,ab,kf.) or ((BME or BAME).ti,ab,kf.) or ("Emigrants and Immigrants"/) or (Refugees/) or (Cultural diversity/) or (Cross Cultural Comparison/) or (exp Human Migration/) or ((refugee* or immigrant* or (asyl* adj1 seek*) or foreign* or ethnic* or minorit* or race or racial* or (multi adj cultural*) or multicultural* or (newly adj arrived) or ((family or families) adj2 reuni*) or resettlement).ti,ab,kf.) or ((cultural or culture or race or racial or nonwhite? or non-white? or (non uk adj (born or national))).ti,ab,kf.) or ((minority adj2 (health* or group?)).ti,ab,kf.) or ((ethnology and utilization).fs.) and (21 or 22 or 23)) or (((Minority Groups/) or (Minority Health/) or (exp Population Groups/) or (exp Ethnic Groups/) or ((ethnic* or multi-ethnic* or multiethnic* or indig* or aborig* or native* or inuit* or eskimo* or kalaallit* or amerind* or romany or romani* or gypsies or

gipsies).ti,ab,kf,hw.) or ((african or africaan or caribbean* or afro-caribbean* or afrocaribbean* or asian* or bangladeshi* or chinese or indian* or pakistani* or somali*).ti,ab,kf,hw.) or ((black or blacks).ti. or (black adj3 (patient? or men or women or adult? or adolesc* or teen* or young* or youth? or old* or elder*)).ti,ab,kf.) or ((BME or BAME).ti,ab,kf.) or ("Emigrants and Immigrants"/) or (Refugees/) or (Cultural diversity/) or (Cross Cultural Comparison/) or (exp Human Migration/) or ((refugee* or immigrant* or (asyl* adj1 seek*) or foreign* or ethnic* or minorit* or race or racial* or (multi adj cultural*) or multicultural* or (newly adj arrived) or ((family or families) adj2 reuni*) or resettlement).ti,ab,kf.) or ((cultural or culture or race or racial or nonwhite? or non-white? or (non uk adj (born or national))).ti,ab,kf.) or ((minority adj2 (health* or group?)).ti,ab,kf.) or ((ethnology and utilization).fs.) and ((Emergency Services, Psychiatric/) or (Hospitals, psychiatric/ and (Crisis intervention/ or emergenc*.ti,kf,hw.)) or ((psychiatric adj3 (in-patients or inpatients or morbidity)).ti,ab,kf.) or (*mental disorders/) or (exp "schizophrenia spectrum and other psychotic disorders"/) or (Forensic Psychiatry/) or ((((mental or psychiatr*) adj (health or disorder* or disease* or deficien* or illness* or problem*)) or psychotic or psychosis or psychoses or psychopath* or schizo* or SMI or bipolar or ((suicid* or self-harm) adj3 (risk or crisis or crises))).ti,ab,kf.) or (((psychiatr* or psychology or health or medicine)) and ((((commit* or admission? or admitt* or detain* or detention? or section* or hospitali#ation?) adj3 (coerc* or compuls* or forced or enforced or involuntary or in-voluntary or rate? or status)).ti,ab,kf.) or (admission?.ab. /freq=2) or (acute admission?.ti,ab,kf.) or (((police or policing or local authority polic*) and (admission? or referral?)).ti,ab,kf,hw.) or (((prison* or custody or incarcerat*) adj3 (transfer* or transition* or referral?)).ti,ab,kf,hw.) or (legal status.ti,ab,kf.) or (*hospitalization/) or (*Hospitals, Psychiatric/ut) or (Psychiatry Department, Hospital/ut) or (coercion/) or ((detention? or admission? or hospitali#ation? or institutionali#ed).ti,kf.) or (((treatment or restriction or court or compulsory) adj order?).ti,ab,kf.) or ((hospitalization/ or patient admission/ or patient discharge/ or patient handoff/ or patient readmission/ or patient transfer/) and ethnology.fs.) or ((Prevalence/ or Incidence/) and (mental health services/ or community mental health services/ or emergency services, psychiatric/) and utilization.fs.) or ((pathway* adj3 (care or hospital* or psychiatr*)).ti,ab,kf.) or (critical pathways/) or (pathway?.ti.) or ("route? to care".ti,ab,kf.) or (exp General Practice/ and diagnosis.fs.) or ((continuity adj3 care).ti,ab,kf. and ((health services or community) and (followup or follow-up or interview* or questionnaire* or focus group*)).ti,ab,kf,hw.) or (conditional* discharge?.ti,ab,kf.) or (revolving door?.ti,ab,kf.) or ((readmission? or re-admission? or recurrence?).ti,kf,hw.)))) and ((randomized controlled trial.pt.) or ((randomi#ed or randomi#ation or randomi#ing).ti,ab,kf.) or ((RCT or "at random" or (random* adj3 (assign* or allocat* or control* or crossover or cross-over or design* or divide* or division or number))).ti,ab,kf.) or ((control* and (placebo or waitlist* or wait* list* or ((treatment or care) adj2 usual) or no intervention* or non intervention* or non-intervention* or without any intervention*)).ti,ab,kf,hw.) or ((control group? or (controlled adj (trial or study))).ti,ab,kf.) or (((single or double) adj blind*).ti,ab,kf,hw.) or (comparative study.pt.))) or (((((Minority Groups/) or (Minority Health/) or (exp Population Groups/) or (exp Ethnic Groups/) or ((ethnic* or multi-ethnic* or multiethnic* or indig* or aborig* or native* or inuit* or eskimo* or kalaallit* or amerind* or romany or romani* or gypsies or gipsies).ti,ab,kf,hw.) or ((african or africaan or caribbean* or afro-caribbean* or afrocaribbean* or asian* or bangladeshi* or chinese or indian* or pakistani* or somali*).ti,ab,kf,hw.) or ((black or blacks).ti. or (black adj3 (patient? or men or women or adult? or adolesc* or teen* or young* or youth? or old* or elder*)).ti,ab,kf.) or ((BME or BAME).ti,ab,kf.) or ("Emigrants and Immigrants"/) or (Refugees/) or (Cultural diversity/) or (Cross Cultural Comparison/) or (exp Human Migration/) or ((refugee* or immigrant* or (asyl* adj1 seek*) or foreign* or ethnic* or minorit* or race or racial* or (multi adj cultural*) or multicultural* or (newly adj arrived) or ((family or families) adj2 reuni*) or resettlement).ti,ab,kf.) or ((cultural or culture or race or racial or nonwhite? or non-white? or (non uk adj (born or national))).ti,ab,kf.) or ((minority adj2 (health* or group?)).ti,ab,kf.) or ((ethnology and utilization).fs.)) and (((mental health adj (act? or law? or legal* or legislat* or jurisprudence)).mp.) or (Commitment of mentally ill/) or ((Mental Disorders/eh or exp Psychotic Disorders/eh) and (psychiatric morbidity.ti,ab,kf. or incidence/ or prevalence/)))) or (((Minority Groups/) or (Minority Health/) or (exp Population Groups/) or (exp Ethnic Groups/) or ((ethnic* or multi-ethnic* or multiethnic* or indig* or aborig* or native* or inuit* or eskimo* or kalaallit* or amerind* or romany or romani* or gypsies or gipsies).ti,ab,kf,hw.) or ((african or africaan or caribbean* or afro-caribbean* or afrocaribbean* or asian* or bangladeshi* or chinese or indian* or pakistani* or somali*).ti,ab,kf,hw.) or ((black or blacks).ti. or (black adj3 (patient? or men or women or adult? or adolesc* or teen* or young* or youth? or old* or elder*)).ti,ab,kf.) or ((BME or BAME).ti,ab,kf.) or ("Emigrants and Immigrants"/) or (Refugees/) or (Cultural diversity/) or (Cross Cultural Comparison/) or (exp Human Migration/) or ((refugee* or immigrant* or (asyl* adj1 seek*) or foreign* or ethnic* or minorit* or race or racial* or (multi adj cultural*) or multicultural* or (newly adj arrived) or ((family or families) adj2 reuni*) or resettlement).ti,ab,kf.) or ((cultural or culture or race or racial or nonwhite? or non-white? or (non uk adj (born or national))).ti,ab,kf.) or ((minority adj2 (health* or group?)).ti,ab,kf.) or ((ethnology and utilization).fs.)) and ((Emergency Services, Psychiatric/) or (Hospitals, psychiatric/ and (Crisis intervention/ or emergenc*.ti,kf,hw.)) or ((psychiatric adj3 (in-patients or inpatients or morbidity)).ti,ab,kf.) or (*mental disorders/) or (exp "schizophrenia spectrum and other psychotic disorders"/) or (Forensic Psychiatry/) or ((((mental or psychiatr*) adj (health or disorder* or disease* or deficien* or illness* or problem*)) or psychotic or psychosis or psychoses or psychopath* or schizo* or SMI or bipolar or ((suicid* or self-harm) adj3 (risk or crisis or crises))).ti,ab,kf.) or (((psychiatr* or psychology or health or

medicine) and (law or legal or legislat* or jurisprudence)).jw.)) and ((commit* or admission? or admitt* or detain* or detention? or section* or hospitali#ation?) adj3 (coerc* or compuls* or forced or enforced or involuntary or in-voluntary or rate? or status)).ti,ab,kf.) or (admission?.ab. /freq=2) or (acute admission?.ti,ab,kf.) or (((police or policing or local authority polic*) and (admission? or referral?)).ti,ab,kf,hw.) or (((prison* or custody or incarcerat*) adj3 (transfer* or transition* or referral?)).ti,ab,kf,hw.) or (legal status.ti,ab,kf.) or (*hospitalization/) or (*Hospitals, Psychiatric/ut) or (Psychiatry Department, Hospital/ut) or (coercion/) or ((detention? or admission? or hospitali#ation? or institutionali#ed).ti,kf.) or (((treatment or restriction or court or compulsory) adj order?).ti,ab,kf.) or ((hospitalization/ or patient admission/ or patient discharge/ or patient handoff/ or patient readmission/ or patient transfer/) and ethnology.fs.) or ((Prevalence/ or Incidence/) and (mental health services/ or community mental health services/ or emergency services, psychiatric/) and utilization.fs.) or ((pathway* adj3 (care or hospital* or psychiatr*)).ti,ab,kf.) or (critical pathways/) or (pathway?.ti.) or ("route? to care".ti,ab,kf.) or (exp General Practice/ and diagnosis.fs.) or ((continuity adj3 care).ti,ab,kf. and ((health services or community) and (followup or follow-up or interview* or questionnaire* or focus group*)).ti,ab,kf,hw.) or (conditional* discharge?.ti,ab,kf.) or (revolving door?.ti,ab,kf.) or ((readmission? or re-admission? or recurrence?).ti,kf,hw.))))) and ((Epidemiologic studies/) or ((epidemiologic* adj3 survey?).ti,ab,kf.) or (exp case control studies/) or (exp cohort studies/) or ((case control or case series or (case adj (records or reports))).ti,ab,kf.) or ((cohort adj (study or studies)).ti,ab,kf.) or (cohort analy$.ti,ab,kf.) or (((followup or follow-up) adj (study or studies)).ti,ab,kf,hw.) or (((followup or follow-up) adj5 (interview* or questionnaire* or focus group*)).ti,ab,kf.) or (((observational or comparative) adj (study or studies)).ti,ab,kf,hw.) or ((prospective or retrospective).ti,ab,kf.) or (longitudinal.ti,ab,kf.) or (cross sectional.ti,ab,kf,hw.) or (((study or research) and (data or analys*)).ti,ab,kf.) or (((qualitative or quantitative) adj (analysis or study or studies)).ti,ab,kf.))) or (((Commitment of mentally ill/) or ((((Emergency Services, Psychiatric/) or (Hospitals, psychiatric/ and (Crisis intervention/ or emergenc*.ti,kf,hw.)) or ((psychiatric adj3 (in-patients or inpatients or morbidity)).ti,ab,kf.) or (*mental disorders/) or (exp "schizophrenia spectrum and other psychotic disorders"/) or (Forensic Psychiatry/) or ((((mental or psychiatr*) adj (health or disorder* or disease* or deficien* or illness* or problem*)) or psychotic or psychosis or psychoses or psychopath* or schizo* or SMI or bipolar or ((suicid* or self-harm) adj3 (risk or crisis or crises))).ti,ab,kf.) or (((psychiatr* or psychology or health or medicine) and (law or legal or legislat* or jurisprudence)).jw.)) and ((((commit* or admission? or admitt* or detain* or detention? or section* or hospitali#ation?) adj3 (coerc* or compuls* or forced or enforced or involuntary or in-voluntary or rate? or status)).ti,ab,kf.) or (coercion/) or ((detention? or admission? or hospitali#ation? or institutionali#ed).ti,kf.) or (((treatment or restriction or court or compulsory) adj order?).ti,ab,kf.))) or (revolving door?.ti,ab,kf.))) and ((meta-analysis.pt. or (meta-analys* or meta analys* or metaanalys* or meta synth* or meta-synth* or metasynth*).ti,ab,kf,hw.) or ((systematic or structured or evidence or trials or studies).ti. and ((review or overview or look or examination or update* or summary).ti. or review.pt.)) or ((0266-4623 or 1469-493X or 1366-5278 or 1530-440X or 2046-4053).is.) or (meta-analysis.pt. or (meta-analys* or meta analys* or metaanalys* or meta synth* or meta-synth* or metasynth*).ti,ab,kf,hw.) or (((systematic or meta) adj2 (analys* or review)).ti,kf. or ((systematic* or quantitativ* or methodologic*) adj5 (review* or overview*)).ti,ab,kf,sh. or (quantitativ$ adj5 synthesis$).ti,ab,kf,hw.) or ((integrative research review* or research integration).tw. or scoping review?.ti,kf. or (review.ti,kf,pt. and (trials as topic or studies as topic).hw.) or (evidence adj3 review*).ti,ab,kf.)))) or (((Minority Groups/) or (Minority Health/) or (exp Population Groups/) or (exp Ethnic Groups/) or ((ethnic* or multi-ethnic* or multiethnic* or indig* or aborig* or native* or inuit* or eskimo* or kalaallit* or amerind* or romany or romani* or gypsies or gipsies).ti,ab,kf,hw.) or ((african or africaan or caribbean* or afro-caribbean* or afrocaribbean* or asian* or bangladeshi* or chinese or indian* or pakistani* or somali*).ti,ab,kf,hw.) or ((black or blacks).ti. or (black adj3 (patient? or men or women or adult? or adolesc* or teen* or young* or youth? or old* or elder*)).ti,ab,kf.) or ((BME or BAME).ti,ab,kf.) or ("Emigrants and Immigrants"/) or (Refugees/) or (Cultural diversity/) or (Cross Cultural Comparison/) or (exp Human Migration/) or ((refugee* or immigrant* or (asyl* adj1 seek*) or foreign* or ethnic* or minorit* or race or racial* or (multi adj cultural*) or multicultural* or (newly adj arrived) or ((family or families) adj2 reuni*) or resettlement).ti,ab,kf.) or ((cultural or culture or race or racial or nonwhite? or non-white? or (non uk adj (born or national))).ti,ab,kf.) or ((minority adj2 (health* or group?)).ti,ab,kf.) or ((ethnology and utilization).fs.)) and ((Emergency Services, Psychiatric/) or (Hospitals, psychiatric/ and (Crisis intervention/ or emergenc*.ti,kf,hw.)) or ((psychiatric adj3 (in-patients or inpatients or morbidity)).ti,ab,kf.) or (*mental disorders/) or (exp "schizophrenia spectrum and other psychotic disorders"/) or (Forensic Psychiatry/) or ((((mental or psychiatr*) adj (health or disorder* or disease* or deficien* or illness* or problem*)) or psychotic or psychosis or psychoses or psychopath* or schizo* or SMI or bipolar or ((suicid* or self-harm) adj3 (risk or crisis or crises))).ti,ab,kf.) or (((psychiatr* or psychology or health or medicine) and (law or legal or legislat* or jurisprudence)).jw.)) and (acute admission?.ti,ab,kf.))

| Ovid PsycINFO 1806 to November Week 2 2017 | | |
|---|---|---|
| # | Searches | Results |
| 1 | ethnology/ or ethnography/ | 9667 |
| 2 | Minority Groups/ | 13352 |
| 3 | exp Ethnic Groups/ | 115331 |
| 4 | exp "racial and ethnic groups"/ | 115331 |
| 5 | ethnic identity/ or multiracial/ | 14571 |
| 6 | "racial and ethnic differences"/ or cross cultural differences/ or cross cultural psychology/ or exp cross cultural treatment/ or multiculturalism/ or multiracial/ or "race and ethnic discrimination"/ | 91506 |
| 7 | Transcultural Psychiatry/ | 980 |
| 8 | (ethnic* or multi-ethnic* or multiethnic* or indig* or aborig* or native* or inuit* or eskimo* or kalaallit* or amerind* or romany or romani* or gypsies or gipsies).ti,ab,id,hw. | 149280 |
| 9 | (african or africaan or caribbean* or afro-caribbean* or afrocaribbean* or asian* or bangladeshi* or chinese or indian* or pakistani* or somali*).ti,ab,id,hw. | 142544 |
| 10 | (black or blacks).ti. or (black adj3 (patient? or men or women or adult? or adolesc* or teen* or young* or youth? or old* or elder*)).ti,ab,id. | 23006 |
| 11 | (BME or BAME).ti,ab,id. | 217 |
| 12 | Refugees/ | 4804 |
| 13 | exp Human Migration/ | 10386 |
| 14 | (refugee* or immigrant* or (asyl* adj1 seek*) or foreign* or ethnic* or minorit* or race or racial* or (multi adj cultural*) or multicultural* or (newly adj arrived) or ((family or families) adj2 reuni*) or resettlement).ti,ab,id. | 210531 |
| 15 | (cultural or culture or race or racial or nonwhite? or non-white? or (non uk adj (born or national))).ti,ab,id. | 309405 |
| 16 | (minority adj2 (health* or group?)).ti,ab,id. | 9020 |
| 17 | *sociocultural factors/ | 28237 |
| 18 | client characteristics/ | 16419 |
| 19 | *demographic characteristics/ | 19573 |
| 20 | or/1-19 | 573586 |
| 21 | exp "Commitment (Psychiatric)"/ | 1764 |
| 22 | (mental health adj2 (act? or law? or legal* or legislat* or jurisprudence)).ti,ab,id. | 2884 |
| 23 | forensic psychology & legal issues.cc. | 16320 |

| | | |
|---|---|---|
| 24 | (psychiatric adj3 (in-patients or inpatients or morbidity)).ti,ab,id. | 11283 |
| 25 | *mental disorders/ or exp Psychosis/ | 165412 |
| 26 | Psychiatric hospitalization/ | 6721 |
| 27 | Psychiatric units/ | 1737 |
| 28 | Psychiatric patients/ | 28078 |
| 29 | Forensic Psychiatry/ | 4241 |
| 30 | *Mental Health Services/ | 27402 |
| 31 | (((mental or psychiatr*) adj (health or disorder* or disease* or deficien* or illness* or problem*)) or psychotic or psychosis or psychoses or psychopath* or schizo* or SMI or bipolar or ((suicid* or self-harm) adj3 (risk or crisis or crises))).ti,ab,id. | 466104 |
| 32 | ((psychiatr* or psychology or health or medicine) and (law or legal or legislat* or jurisprudence)).jw. | 8758 |
| 33 | or/21-32 | 521800 |
| 34 | ((commit* or admission? or admitt* or detain* or detention? or section* or hospitali#ation?) adj3 (coerc* or compuls* or forced or enforced or involuntary or in-voluntary or rate? or status)).ti,ab,id. | 6644 |
| 35 | ((admit* or admission* or detain* or detention or hospitali#ation) adj3 section).ti,ab,id. | 75 |
| 36 | exp legal detention/ | 665 |
| 37 | Court referrals/ | 694 |
| 38 | exp Involuntary Treatment/ or assertive community treatment/ | 1713 |
| 39 | admission?.ab. /freq=2 | 10603 |
| 40 | acute admission?.ti,ab,id. | 197 |
| 41 | ((inpatient? or in-patient?) adj2 admission?).ti,ab,id. | 1130 |
| 42 | ((police or policing or local authority polic*) and (admission? or referral?)).ti,ab,id,hw. | 640 |
| 43 | ((prison* or custody or incarcerat*) adj3 (transfer* or transition* or referral?)).ti,ab,id,hw. | 307 |
| 44 | exp *Legislative Processes/ | 1886 |
| 45 | legal status.ti,ab,id. | 1211 |
| 46 | *laws/ or *legal processes/ | 21000 |
| 47 | *hospitalization/ | 4635 |
| 48 | high-security.ti,ab,id. | 449 |
| 49 | coercion.ti,ab,id,hw. | 4879 |
| 50 | (detention? or admission? or hospitali#ation? or institutionali#ed).ti,id. | 20392 |
| 51 | ((treatment or restriction or court or compulsory) adj order?).ti,ab,id. | 833 |

| | | |
|---|---|---|
| 52 | (pathway* adj3 (care or hospital* or psychiatr*)).ti,ab,id. | 1237 |
| 53 | pathway?.ti. | 12275 |
| 54 | "route? to care".ti,ab,id. | 15 |
| 55 | ((outcome? or course or predictor?) and first-episode).ti,id,hw. | 767 |
| 56 | (continuity adj3 care).ti,ab,id. and ((health services or community) and (followup or follow-up or interview* or questionnaire* or focus group*)).ti,ab,id,hw. | 240 |
| 57 | conditional* discharge*.ti,ab,id. | 56 |
| 58 | revolving door?.ti,ab,id. | 355 |
| 59 | (readmission? or re-admission? or recurrence?).ti,id,hw. | 3291 |
| 60 | (*diagnosis/ or *psychodiagnosis/) and *general practitioners/ | 173 |
| 61 | utili#ation.ti,id,hw. and (differ* or compar* or prevalence or incidence).ti. | 1514 |
| 62 | or/34-61 | 81025 |
| 63 | 20 and 33 and 62 | 3996 |
| 64 | (Systematic Review or Meta Analysis or Metasynthesis).ti,md,sh. | 36533 |
| 65 | 33 and 62 and 64 | 191 |
| 66 | 63 or 65 | 4162 |
| 67 | (afrikaans or albanian or arabic or bulgarian or catalan or chinese or croatian or czech or danish or dutch or estonian or farsi iranian or finnish or french or georgian or german or greek or hebrew or hindi or hungarian or italian or japanese or korean or lithuanian or malaysian or nonenglish or norwegian or polish or portuguese or romanian or russian or serbian or serbo croatian or slovak or slovene or spanish or swedish or turkish or ukrainian or urdu).lg. | 308068 |
| 68 | 66 not 67 | 3894 |
| 69 | mental health act.id. | 321 |
| 70 | *"commitment (psychiatric)"/ | 1354 |
| 71 | (*Mental Health Services/ or *Mental Disorders/) and Inpatient & Hospital Services.cc. | 2274 |
| 72 | *"Racial and Ethnic Differences"/ and Health & Mental Health Services.cc. and utili#ation.mp. | 262 |
| 73 | (*"Racial and Ethnic Differences"/ or exp "racial and ethnic groups"/) and (*"Psychiatric Hospital Admission"/ or *Psychiatric Hospitalization/) | 118 |
| 74 | 69 or (21 and (70 or 71)) or 72 or 73 | 1186 |
| 75 | (empirical study or followup study or longitudinal study or meta analysis or metasynthesis or prospective study or qualitative study or quantitative study or retrospective study or "systematic review" or treatment outcome).md. | 2E+06 |
| 76 | (74 and 75) not (67 or 68) | 425 |
| 77 | (68 or 76) | 4319 |

| Embase 1974 to 2017 Week 47 | | |
|---|---|---|
| # | Searches | Results |
| 1 | ethnology/ or ethnography/ | 73575 |
| 2 | exp ancestry group/ | 244889 |
| 3 | Minority Group/ | 13505 |
| 4 | exp Ethnic Group/ | 119954 |
| 5 | "ethnic or racial aspects"/ or cultural factor/ or ethnic difference/ or ethnicity/ or race/ or race difference/ | 220166 |
| 6 | cross cultural differences/ or multiculturalism/ | 146 |
| 7 | Transcultural Psychiatry/ | 101 |
| 8 | (ethnic* or multi-ethnic* or multiethnic* or indig* or aborig* or native* or inuit* or eskimo* or kalaallit* or amerind* or romany or romani* or gypsies or gipsies).ti,ab,kw,hw. | 521368 |
| 9 | (african or africaan or caribbean* or afro-caribbean* or afrocaribbean* or asian* or bangladeshi* or chinese or indian* or pakistani* or somali*).ti,ab,kw,hw. | 666162 |
| 10 | (black or blacks).ti. or (black adj3 (patient? or men or women or adult? or adolesc* or teen* or young* or youth? or old* or elder*)).ti,ab,kw. | 52900 |
| 11 | (BME or BAME).ti,ab,kw. | 1739 |
| 12 | Refugee/ | 9955 |
| 13 | exp Human Migration/ | 42366 |
| 14 | (refugee* or immigrant* or (asyl* adj1 seek*) or foreign* or ethnic* or minorit* or race or racial* or (multi adj cultural*) or multicultural* or (newly adj arrived) or ((family or families) adj2 reuni*) or resettlement).ti,ab,kw. | 437376 |
| 15 | (cultural or culture or race or racial or nonwhite? or non-white? or (non uk adj (born or national))).ti,ab,kw. | 901607 |
| 16 | (minority adj2 (health* or group?)).ti,ab,kw. | 7801 |
| 17 | or/1-16 | 2207129 |
| 18 | (mental health adj2 (act? or law? or legal* or legislat* or jurisprudence)).ti,ab,kw. | 2621 |
| 19 | (psychiatric adj3 (in-patients or inpatients or morbidity)).ti,ab,kw. | 12708 |
| 20 | psychiatric.ti,kw,hw. and hospital patient/ | 5103 |
| 21 | *Mental Hospital/ or mental patient/ | 36793 |
| 22 | *mental health/ or *mental disease/ or exp Psychosis/ | 369183 |
| 23 | *Psychiatric department/ | 2902 |
| 24 | Mental Health Services/ | 47821 |

| | | |
|---|---|---|
| 25 | (((mental or psychiatr*) adj (health or disorder* or disease* or deficien* or illness* or problem*)) or psychotic or psychosis or psychoses or psychopath* or schizo* or SMI or bipolar or ((suicid* or self-harm) adj3 (risk or crisis or crises))).ti,ab,kw. | 542104 |
| 26 | or/18-25 | 701161 |
| 27 | involuntary commitment/ | 1019 |
| 28 | mandatory program/ | 2206 |
| 29 | Forensic Psychiatry/ | 12855 |
| 30 | ((psychiatr* or psychology or health or medicine) and (law or legal or legislat* or jurisprudence)).jw. | 33774 |
| 31 | ((commit* or admission? or admitt* or detain* or detention? or section* or hospitali#ation?) adj3 (coerc* or compuls* or forced or enforced or involuntary or in-voluntary or rate? or status)).ti,ab,kw. | 41307 |
| 32 | ((admit* or admission* or detain* or detention or hospitali#ation) adj3 section).ti,ab,kw. | 331 |
| 33 | exp Involuntary Treatment/ or assertive community treatment/ | 1107 |
| 34 | admission?.ab. /freq=2 | 107392 |
| 35 | acute admission?.ti,ab,kw. | 1266 |
| 36 | hospital admission/ | 159682 |
| 37 | ((inpatient? or in-patient?) adj2 admission?).ti,ab,kw. | 7458 |
| 38 | ((police or policing or local authority polic*) and (admission? or referral?)).ti,ab,kw,hw. | 1114 |
| 39 | ((prison* or custody or incarcerat*) adj3 (transfer* or transition* or referral?)).ti,ab,kw,hw. | 273 |
| 40 | patient referral/ and legal aspect/ | 1833 |
| 41 | legal status.ti,ab,kw. | 1722 |
| 42 | high-security.ti,ab,kw. | 709 |
| 43 | coercion.ti,ab,kw,hw. | 3374 |
| 44 | persuasive communication/ | 7707 |
| 45 | (detention? or admission? or hospitali#ation? or institutionali#ed).ti,kw. | 56852 |
| 46 | ((treatment or restriction or court or compulsory) adj order?).ti,ab,kw. | 1451 |
| 47 | (pathway* adj3 (care or hospital* or psychiatr*)).ti,ab,kw. | 7907 |
| 48 | clinical pathway/ | 7565 |
| 49 | pathway?.ti. | 203348 |
| 50 | "route? to care".ti,ab,kw. | 92 |
| 51 | ((outcome? or course or predictor?) and first-episode).ti,kw,hw. | 1785 |

| | | |
|---|---|---|
| 52 | (continuity adj3 care).ti,ab,kw. and ((health services or community) and (followup or follow-up or interview* or questionnaire* or focus group*)).ti,ab,kw,hw. | 977 |
| 53 | conditional* discharge*.ti,ab,kw. | 43 |
| 54 | revolving door?.ti,ab,kw. | 410 |
| 55 | (readmission? or re-admission? or recurrence?).ti,kw,hw. | 302978 |
| 56 | (*diagnosis/ or *psychodiagnosis/) and *general practitioners/ | 98 |
| 57 | utili#ation.ti,kw,hw. and (differ* or compar* or prevalence or incidence).ti. | 11142 |
| 58 | health care utilization/ | 58298 |
| 59 | or/27-58 | 902113 |
| 60 | 17 and 26 and 59 | 5742 |
| 61 | psychiatry.ec. | 961785 |
| 62 | 60 and 61 | 3210 |
| 63 | exp evidence based medicine/ | 1054763 |
| 64 | (RCT or random* or prospective or retrospective or outcome?).mp. | 4665653 |
| 65 | (study or trial?).ti,hw. | 1.2E+07 |
| 66 | (case control or case series or (case adj (records or reports))).ti,ab,kw. | 289592 |
| 67 | ((followup or follow-up) adj5 (interview* or questionnaire* or focus group*)).ti,ab,kw. | 16182 |
| 68 | (longitudinal or cross-sectional).ti,ab,kw,hw. | 665229 |
| 69 | (cohort adj (analysis or study or studies)).ti,ab,kw,hw. | 396513 |
| 70 | ((control* or comparative or follow-up or followup or observational or epidemiologic*) adj (study or studies)).mp. | 6800837 |
| 71 | ((qualitative or quantitative) adj (analysis or study or studies)).ti,ab,kw. | 115818 |
| 72 | ((study or research) and (data or analys*)).ti,ab,kw. | 3942145 |
| 73 | or/63-72 | 1.5E+07 |
| 74 | (systematic or structured or evidence or trials or studies).ti. and ((review or overview or look or examination or update* or summary).ti. or review.sh.) | 166828 |
| 75 | (0266-4623 or 1469-493X or 1366-5278 or 1530-440X or 2046-4053).is. | 21782 |
| 76 | (meta-analys* or meta analys* or metaanalys* or meta synth* or meta-synth* or metasynth*).ti,ab,kw. | 161073 |
| 77 | (integrative research review* or research integration).ti,ab,kw. or scoping review?.ti,kw. or (review.ti,kw,sh. and (trials or studies).hw.) or (evidence adj3 review*).ti,ab,kw. | 49041 |
| 78 | ((systematic or meta) adj2 (analys* or review)).ti,kw. or ((systematic* or quantitativ* or methodologic*) adj5 (review* or overview*)).ti,ab,kw,sh. or (quantitativ$ adj5 synthesis$).ti,ab,kw,hw. | 269795 |

| | | |
|---|---|---|
| 79 | review.sh. and ((medline or medlars or embase or pubmed or scisearch or psychinfo or psycinfo or psychlit or psyclit or cinahl or electronic database* or bibliographic database* or computeri#ed database* or online database* or pooling or pooled or mantel haenszel or peto or dersimonian or der simonian or fixed effect or ((hand adj2 search*) or (manual* adj2 search*))).ti,ab,kw,hw. or retracted article.sh.) | 95806 |
| 80 | or/74-79 | 442877 |
| 81 | 62 and (73 or 80) | 2493 |
| 82 | (27 or (26 and (31 or 43 or 44 or 45 or 46))) and 80 | 299 |
| 83 | 81 or 82 | 2764 |
| 84 | limit 83 to (article-in-press status or embase status or in-process status) | 2676 |
| 85 | (editorial or letter or note).pt. | 2251003 |
| 86 | (afrikaans or albanian or arabic or armenian or azerbaidzhani or basque or belorussian or bosnian or bulgarian or catalan or chinese or croatian or czech or danish or dutch or esperanto or estonian or finnish or french or gallegan or georgian or german or greek or hebrew or hindi or hungarian or icelandic or indonesian or irish gaelic or italian or japanese or korean or latvian or lithuanian or macedonian or malay or norwegian or persian or polish or polyglot or portuguese or pushto or romanian or russian or serbian or slovak or slovene or spanish or swedish or thai or turkish or ukrainian or urdu or uzbek or vietnamese).lg. | 5408326 |
| 87 | 84 not (85 or 86) | **2515** |

**Search Name: UCL-MHA**
**Last Saved: 27/11/2017 22:55:30.775**
**Description: The Cochrane Library, Issue 10, 2017**

| ID | Search | |
|---|---|---|
| #1 | MeSH descriptor: [Minority Groups] this term only | |
| #2 | MeSH descriptor: [Minority Health] this term only | |
| #3 | MeSH descriptor: [Population Groups] explode all trees | |
| #4 | MeSH descriptor: [Ethnic Groups] explode all trees | |
| #5 | (ethnic* or multi-ethnic* or multiethnic* or indig* or aborig* or native* or inuit* or eskimo* or kalaallit* or amerind* or romany or romani* or gypsies or gipsies):ti,ab,kw (Word variations have been searched) | |
| #6 | (african or africaan or caribbean* or afro-caribbean* or afrocaribbean* or asian* or bangladeshi* or chinese or indian* or pakistani* or somali*):ti,ab,kw (Word variations have been searched) | |
| #7 | (black near/3 (patient* or men or women or adult* or adolesc* or teen* or young* or youth* or old* or elder*)):ti,ab,kw (Word variations have been searched) | |
| #8 | (black or blacks):ti | |
| #9 | (BME or BAME):ti,ab,kw (Word variations have been searched) | |
| #10 | MeSH descriptor: [Emigrants and Immigrants] explode all trees | |
| #11 | MeSH descriptor: [Refugees] this term only | |
| #12 | MeSH descriptor: [Cultural Diversity] this term only | |
| #13 | MeSH descriptor: [Cross-Cultural Comparison] this term only | |
| #14 | MeSH descriptor: [Human Migration] explode all trees | |
| #15 | (refugee* or immigrant* or (asyl* near/2 seek*) or foreign* or ethnic* or minorit* or race or racial* or (multi next cultural*) or multicultural* or (newly next arrived) or ((family or families) near/2 reuni*) or resettlement):ti,ab,kw (Word variations have been searched) | |
| #16 | (cultural or culture or race or racial or nonwhite* or non-white* or ("non uk" next (born or national))):ti,ab,kw (Word variations have been searched) | |
| #17 | (minority near/2 (health* or group*)):ti,ab,kw (Word variations have been searched) | |
| #18 | #1 or #2 or #3 or #4 or #5 or #6 or #7 or #8 or #9 or #10 or #11 or #12 or #13 or #14 or #15 or #16 or #17 | |
| #19 | "Mental Health Act":ti,ab,kw (Word variations have been searched) | |
| #20 | MeSH descriptor: [Commitment of Mentally Ill] this term only | |
| #21 | ("mental health" next (law* or legal* or legislat* or jurisprudence)):ti,ab,kw (Word variations have been searched) | |
| #22 | MeSH descriptor: [Mental Disorders] explode all trees and with qualifier(s): [Ethnology - EH] | |

| | | |
|---|---|---|
| #23 | MeSH descriptor: [Psychotic Disorders] explode all trees and with qualifier(s): [Ethnology - EH] | |
| #24 | #19 or #20 or #21 or #22 or #23 | |
| #25 | MeSH descriptor: [Emergency Services, Psychiatric] this term only | |
| #26 | MeSH descriptor: [Hospitals, Psychiatric] this term only | |
| #27 | (psychiatric near/3 (in-patients or inpatients or morbidity)):ti,ab,kw (Word variations have been searched) | |
| #28 | MeSH descriptor: [Mental Disorders] this term only | |
| #29 | MeSH descriptor: [Schizophrenia Spectrum and Other Psychotic Disorders] explode all trees | |
| #30 | MeSH descriptor: [Forensic Psychiatry] this term only | |
| #31 | (((mental or psychiatr*) next (health or disorder* or disease* or deficien* or illness* or problem*)) or psychotic or psychosis or psychoses or psychopath* or schizo* or SMI or bipolar or ((suicid* or self-harm) near/3 (risk or crisis or crises))):ti,ab,kw (Word variations have been searched) | |
| #32 | ((psychiatr* or psychology or health or medicine) and (law or legal or legislat* or jurisprudence)):so (Word variations have been searched) | |
| #33 | #25 or #26 or #27 or #28 or #29 or #30 or #31 or #32 | |
| #34 | ((commit* or admission* or admitt* or detain* or detention* or section* or hospitalisation* or hospitalization*) near/3 (coerc* or compuls* or forced or enforced or involuntary or in-voluntary or rate or rates or status)):ti,ab,kw (Word variations have been searched) | |
| #35 | admission*:ti,ab,kw | |
| #36 | ((prison* or custody or incarcerat*) near/3 (transfer* or transition* or referral*)):ti,ab,kw (Word variations have been searched) | |
| #37 | "legal status":ti,ab,kw (Word variations have been searched) | |
| #38 | MeSH descriptor: [Hospitalization] explode all trees | |
| #39 | MeSH descriptor: [Hospitals, Psychiatric] this term only | |
| #40 | MeSH descriptor: [Psychiatric Department, Hospital] this term only | |
| #41 | MeSH descriptor: [Coercion] this term only | |
| #42 | (detention* or admission* or hospitalization* or hospitalisation* or institutionali*):ti (Word variations have been searched) | |
| #43 | ((treatment or restriction or court or compulsory) next order*):ti,ab,kw (Word variations have been searched) | |
| #44 | MeSH descriptor: [Morbidity] explode all trees | |
| #45 | MeSH descriptor: [Mental Health Services] explode all trees | |
| #46 | #44 and #45 | |
| #47 | pathway* or "route to care":ti,ab,kw (Word variations have been searched) | |
| #48 | MeSH descriptor: [Critical Pathways] this term only | |

| | | |
|---|---|---|
| #49 | "conditional* discharge*":ti,ab,kw (Word variations have been searched) | |
| #50 | "revolving door*":ti,ab,kw (Word variations have been searched) | |
| #51 | (readmission* or re-admission* or recurrence*):ti,ab,kw (Word variations have been searched) | |
| #52 | ("health services" or community) and (followup or follow-up or interview* or questionnaire* or focus group*):ti,ab,kw (Word variations have been searched) | |
| #53 | #34 or #35 or #36 or #37 or #38 or #39 or #40 or #41 or #42 or #43 or #46 or #47 or #48 or #49 or #50 or #51 or #52 | |
| #54 | #18 and #33 and #53 | |
| #55 | #24 or #54 | 1018 (Trials) |
| | | 52 (Reviews) |
| | Total | 1070 records |

**Interface - EBSCOhost Research Databases**
**Search Screen - Advanced Search**
**Database - CINAHL (1981 to 30 November 2017)**

| # | Searches | Results |
|---|---|---|
| S75 | S73 OR S74 | 1,671 |
| S74 | S17 AND S70 | 82 |
| S73 | S70 AND S71 AND S72 | 1,644 |
| S72 | S33 OR S34 OR S35 OR S36 OR S37 OR S38 OR S39 OR S40 OR S41 OR S42 OR S43 OR S44 OR S45 OR S46 OR S47 OR S48 OR S49 OR S50 OR S51 OR S52 OR S53 OR S54 OR S55 | 204,561 |
| S71 | S18 OR S19 OR S20 OR S21 OR S22 OR S23 OR S24 OR S25 OR S26 OR S27 OR S28 OR S29 OR S30 OR S31 OR S32 | 193,736 |

| | | |
|---|---|---|
| S70 | S1 OR S2 OR S3 OR S4 OR S5 OR S6 OR S7 OR S8 OR S9 OR S10 OR S11 OR S12 OR S13 OR S14 | 178,704 |
| S69 | (evidence N3 review) or ("integrative research review*" or "research integration") | |
| S68 | ((systematic* or quantitativ* or methodologic*) N5 (review* or overview* or synthes*)) | |
| S67 | (meta-analys* or meta analys* or metaanalys* or meta synth* or meta-synth* or metasynth*) | |
| S66 | (MH "Meta Analysis") | |
| S65 | (MH "Systematic Review") OR (MH "Scoping Review") | |
| S64 | ((study or research) and (data or analys*)) | |
| S63 | ((qualitative or quantitative) N1 (analysis or study or studies)) | |
| S62 | ((control* or comparative or follow-up or followup or observational or epidemiologic*) N1 (study or studies)) | |
| S61 | (cohort N1 (analysis or study or studies)) | |
| S60 | longitudinal or cross-sectional | |
| S59 | ((followup or follow-up) N5 (interview* or questionnaire*)) or "focus group*" | |
| S58 | ("case control" or "case series" or (case N1 (records or reports))) | |
| S57 | TI (study or trial or trials) | |
| S56 | (RCT or random* or prospective or retrospective or outcome*) | |
| S55 | (MM "Morbidity") OR (MM "Incidence") OR (MM "Prevalence") | |
| S54 | (MH "Health Resource Utilization") | |
| S53 | TI (readmission* or re-admission* or readmit* or re-admit* or recurrence*) | |
| S52 | "revolving door*" | |
| S51 | "conditional* discharge*" | |
| S50 | (followup or follow-up) | |
| S49 | (continuity N3 care) | |
| S48 | ((outcome* or course or predictor*) and first-episode) | |
| S47 | "route* to care" | |
| S46 | TI pathway* | |
| S45 | (MH "Critical Path") | |
| S44 | (pathway* N3 (care or hospital* or psychiatr*)) | |
| S43 | ((treatment or restriction or court or compulsory) N1 order*) | |
| S42 | coercion | |
| S41 | "high security" | |
| S40 | "legal status" | |
| S39 | TI detention* or admission* or hospitalization* or hospitalisation* or institutionali*) | |
| S38 | (MH "Coercion") | |
| S37 | ((prison* or custody or incarcerat*) N3 (transfer* or transition* or referral*)) | |
| S36 | ((police or policing or "local authority polic*") and (admission* or referral*)) | |
| S35 | ((acute or hospital* or inpatient* or in-patient*) N2 admission*) | |

| | | |
|---|---|---|
| S34 | (MH "Hospitalization") OR (MH "Length of Stay") OR (MH "Patient Admission") OR (MH "Readmission") OR (MH "Institutionalization") | |
| S33 | ((commit* or admission* or admitt* or detain* or detention* or section* or hospitalization* or hospitalisation*) N3 (coerc* or compuls* or forced or enforced or involuntary or in-voluntary or rate or rates or status)) | |
| S32 | SO ((psychiatr* or psychology or health or medicine) and (law or legal or legislat* or jurisprudence)) | |
| S31 | (MH "Forensic Psychiatry+") | |
| S30 | (((mental or psychiatr*) N1 (health or disorder* or disease* or deficien* or illness* or problem*)) or psychotic or psychosis or psychoses or psychopath* or schizo* or SMI or bipolar) | |
| S29 | (psychiatric N3 (in-patients or inpatients or morbidity) | |
| S28 | ("mental health" N2 (act* or law* or legal* or legislat* or jurisprudence)) | |
| S27 | (MH "Psychiatric Emergencies") | |
| S26 | (MH "Emergency Services, Psychiatric") | |
| S25 | (MH "Community Mental Health Nursing") | |
| S24 | (MH "Psychiatric Nursing+") | |
| S23 | (MH "Hospitals, Psychiatric") | |
| S22 | (MH "Psychiatric Care+") | |
| S21 | (MH "Psychiatric Patients+") | |
| S20 | (MM "Mental Health") | |
| S19 | (MM "Mental Disorders") OR (MH "Personality Disorders+") OR (MH "Psychotic Disorders+") | |
| S18 | (MH "Mental Health Services+/LJ/TD/PF/UT") | |
| S17 | S15 OR S16 | |
| S16 | (MH "Involuntary Commitment") | |
| S15 | "Mental Health Act" | |
| S14 | (MH "Ethnology") OR (MH "Ethnological Research") | |
| S13 | (minority N2 (health* or group*)) | |
| S12 | ((cultural or culture or race or racial) N2 (compar* or differ* or disparit* or discriminat* or survey* or stratif*)) or nonwhite* or non-white* or ("non uk" N2 (born or national*)) | |
| S11 | (refugee* or immigrant* or (asyl* N1 seek*) or ethnic* or "multi cultural*" or multicultural*) | |
| S10 | TI ( BME or BAME ) OR AB ( BME or BAME ) | |
| S9 | (black N3 (patient* or men or women or adult* or adolesc* or teen* or young* or youth* or old* or elder*)) | |
| S8 | TI black or blacks or negro or negroes | |
| S7 | (african or africaan or caribbean* or afro-caribbean* or afrocaribbean* or asian* or bangladeshi* or chinese or indian* or pakistani* or somali*) | |
| S6 | (ethnic* or multi-ethnic* or multiethnic* or indig* or aborig* or inuit* or eskimo* or kalaallit* or amerind* or romany or romani* or gypsies or gipsies) | |

| | | |
|---|---|---|
| S5 | (MH "Refugees") | |
| S4 | (MH "Immigrants+") | |
| S3 | (MH "Race Factors") | |
| S2 | (MH "Ethnic Groups+") OR (MM "Culture+") | |
| S1 | (MH "Minority Groups") | |

**Funnel plots for all analyses**

**Black African**

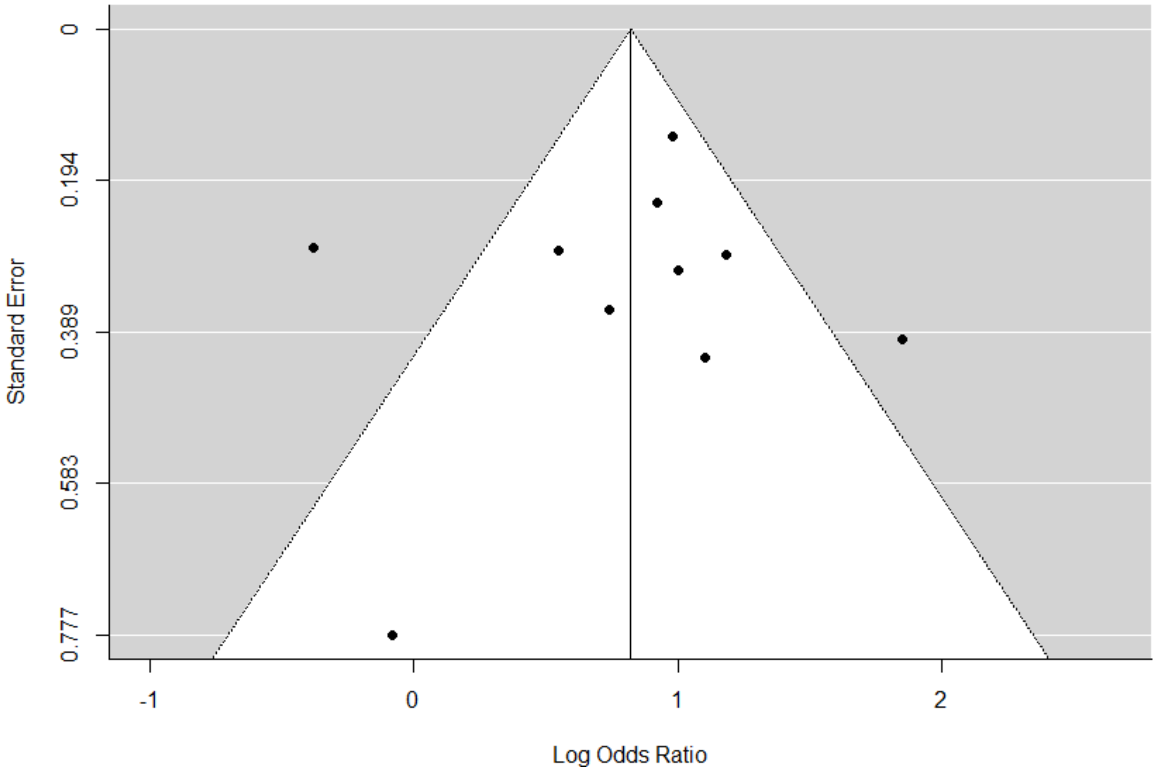


**Black Caribbean**

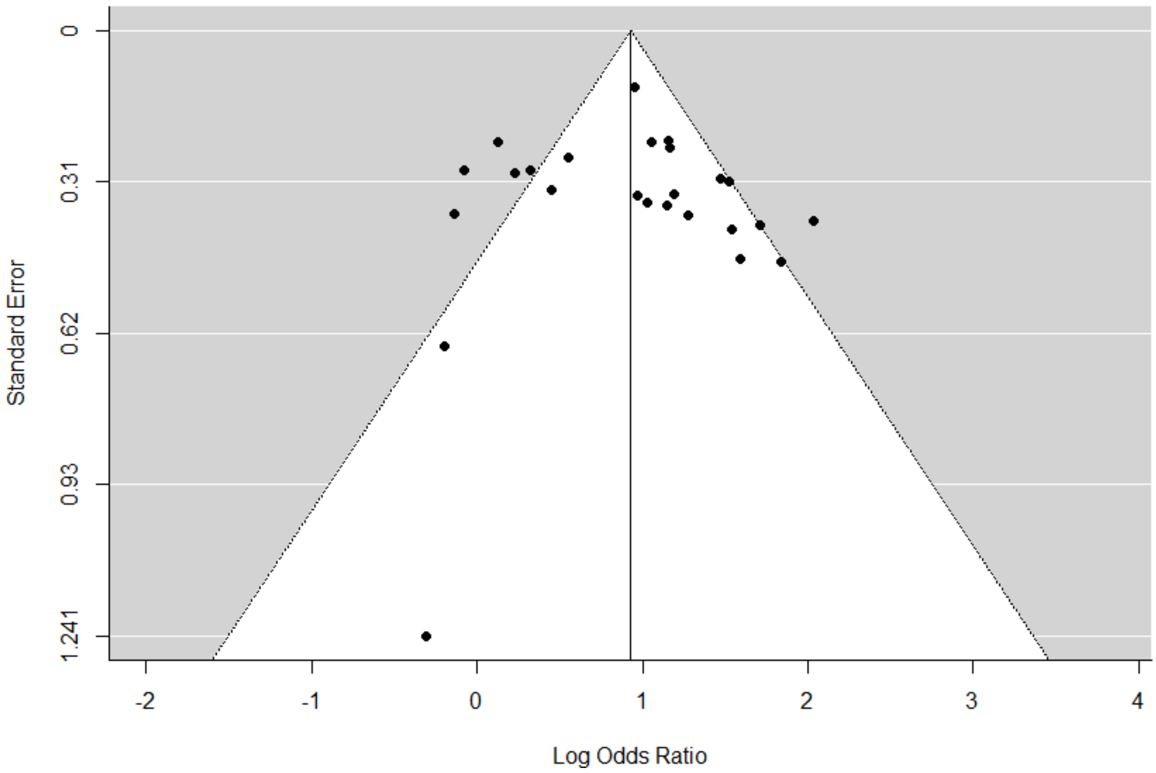

**Black Unspecified**

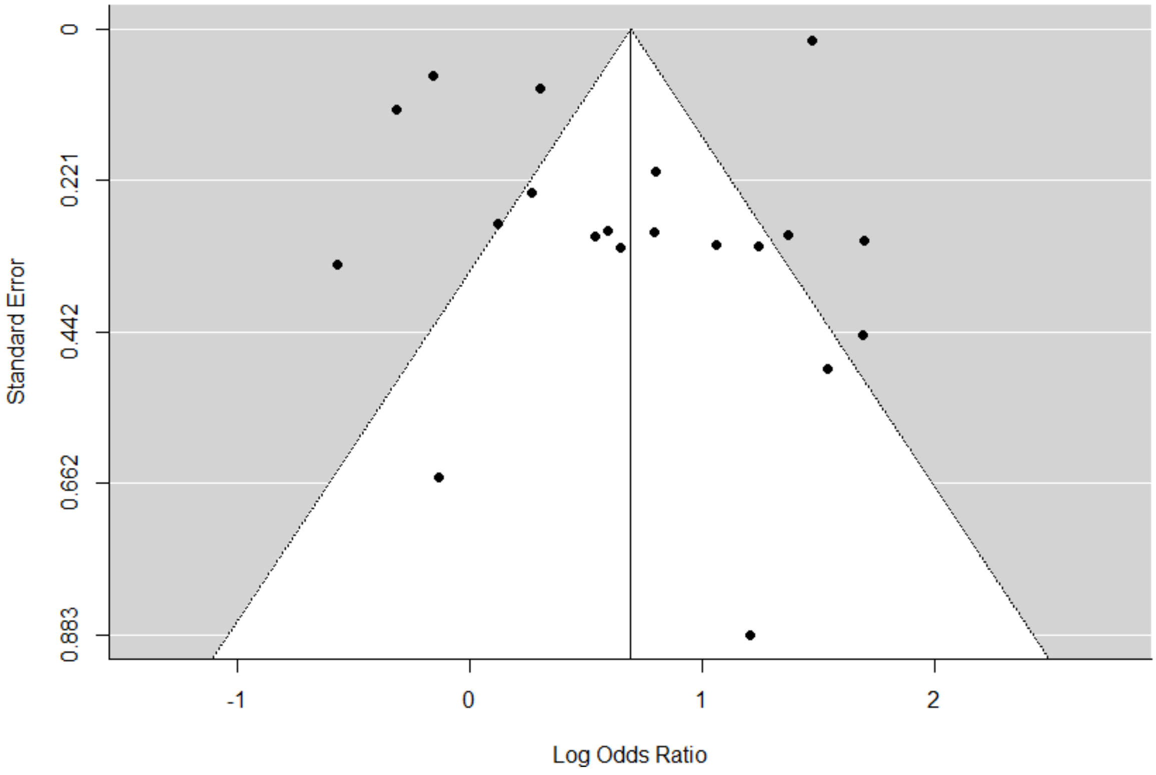


**South Asian**

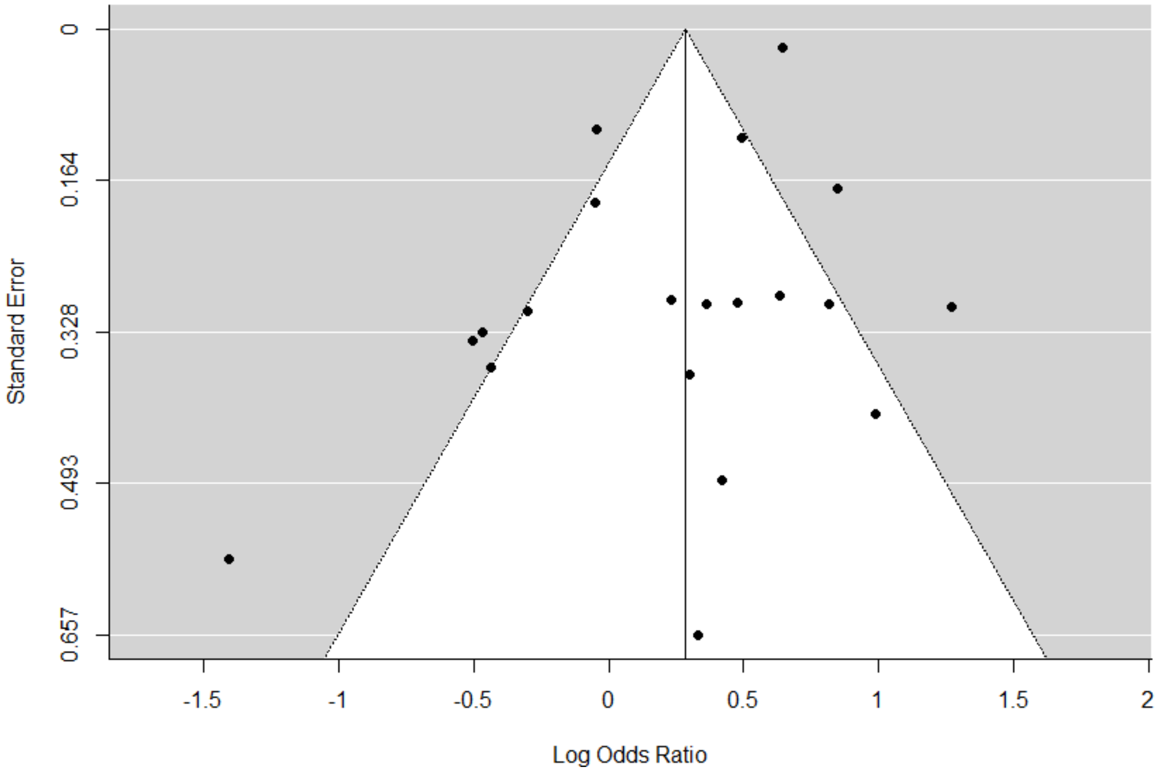

**East Asian**

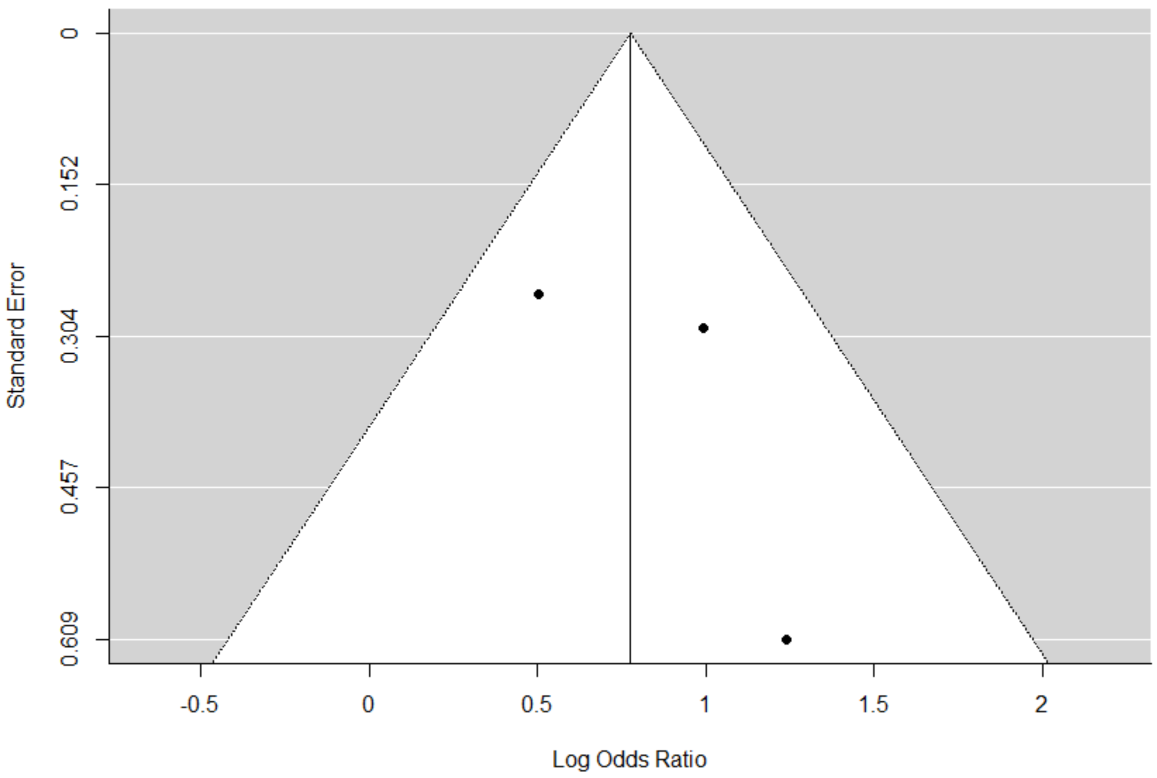


**Immigrants**

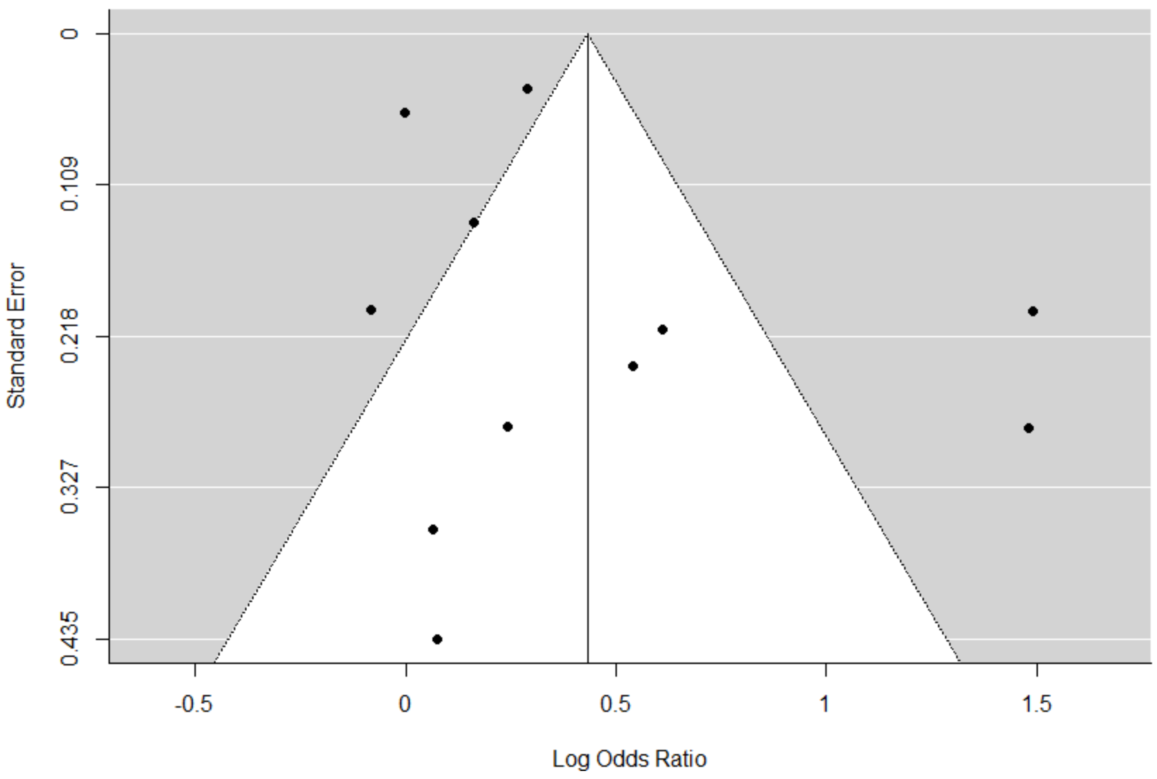

**Mixed BAME**

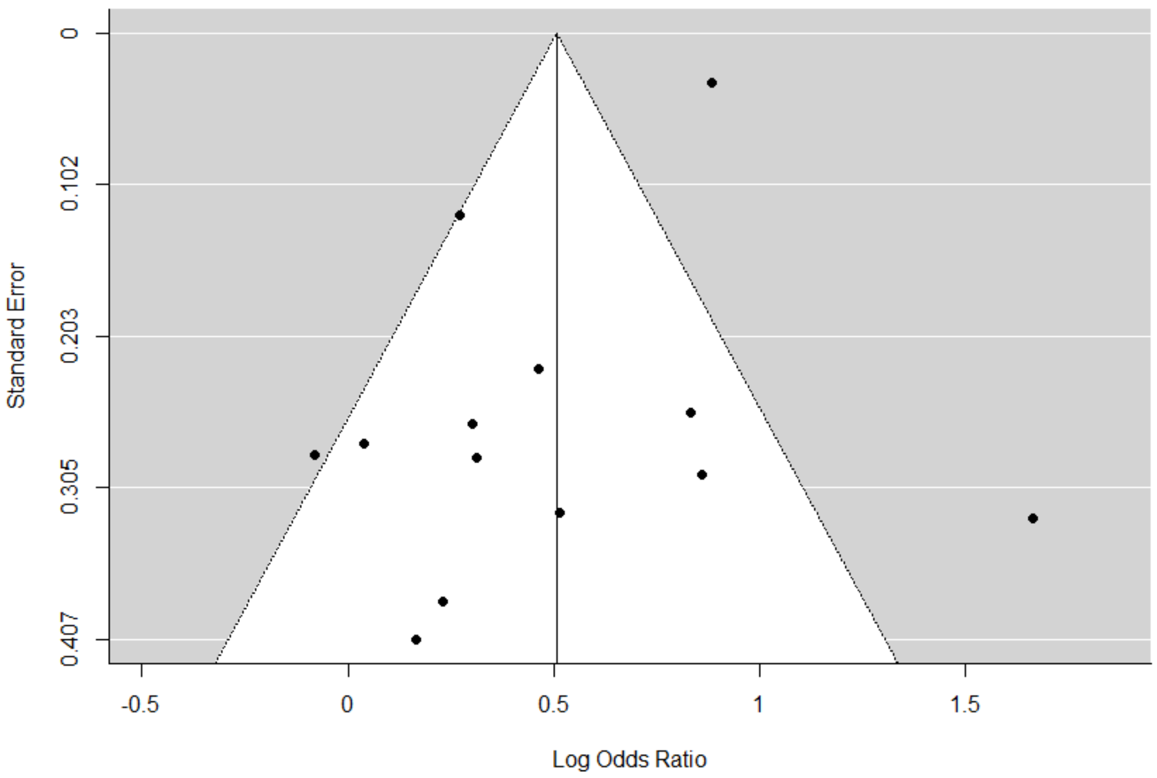

0
0.102
0.203
0.305
0.407
Standard Error
-0.5
0
0.5
1
1.5
Log Odds Ratio

**Forest plots for all analyses.**

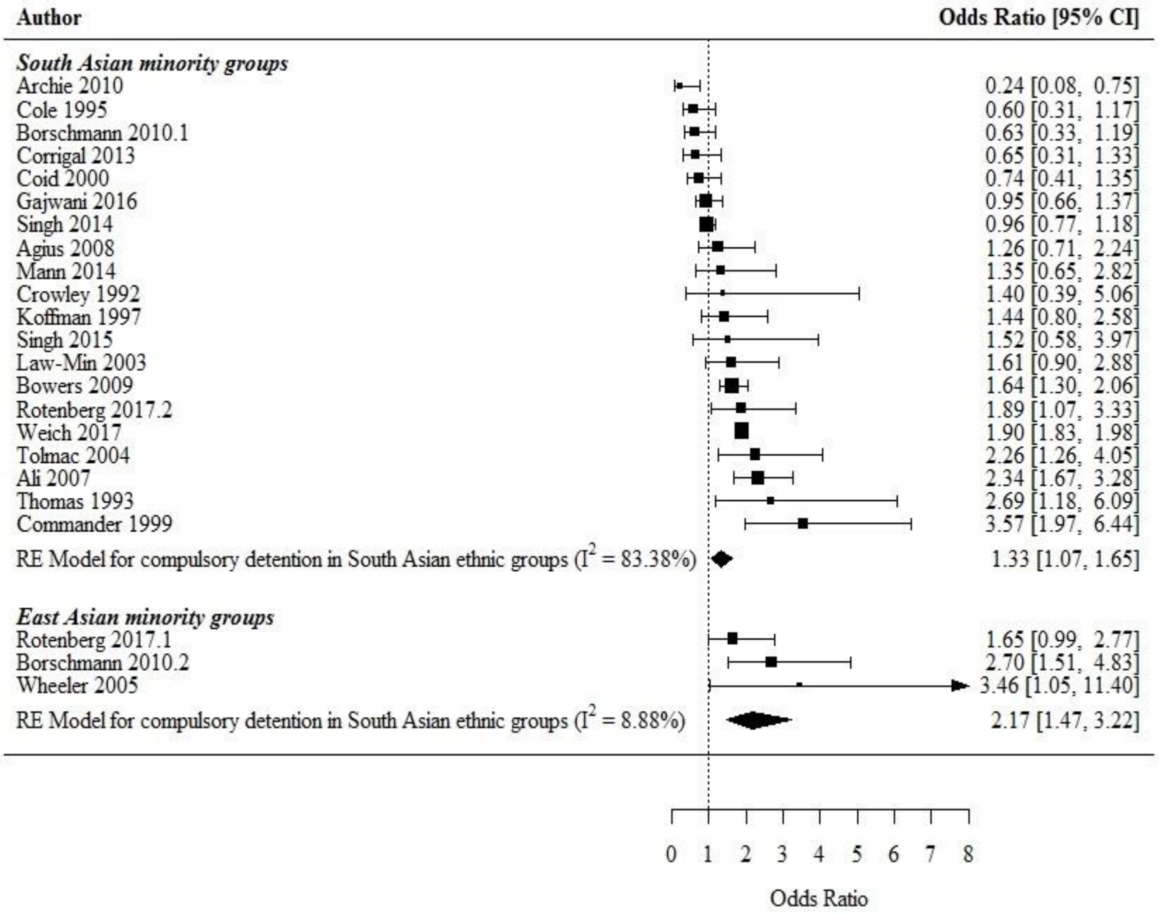

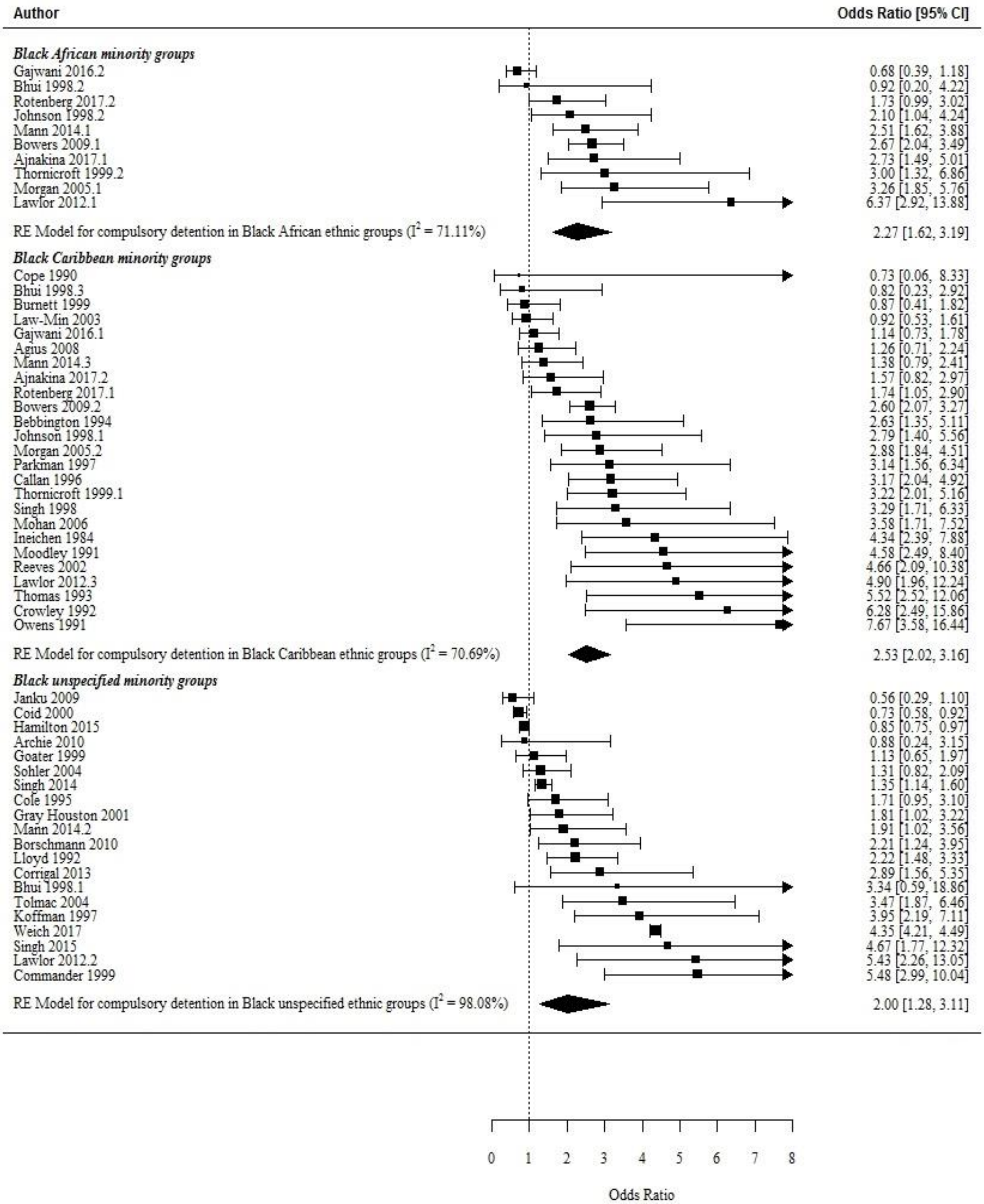
Author
Odds Ratio [95% CI]
Black African minority groups
Gajwani 2016.2 0.68 [0.39, 1.18]
Bhui 1998.2 0.92 [0.20, 4.22]
Rotenberg 2017.2 1.73 [0.99, 3.02]
Johnson 1998.2 2.10 [1.04, 4.24]
Mann 2014.1 2.51 [1.62, 3.88]
Bowers 2009.1 2.67 [2.04, 3.49]
Ajnakina 2017.1 2.73 [1.49, 5.01]
Thornicroft 1999.2 3.00 [1.32, 6.86]
Morgan 2005.1 3.26 [1.85, 5.76]
Lawlor 2012.1 6.37 [2.92, 13.88]
RE Model for compulsory detention in Black African ethnic groups ($I^2$ = 71.11%) 2.27 [1.62, 3.19]
Black Caribbean minority groups
Cope 1990 0.73 [0.06, 8.33]
Bhui 1998.3 0.82 [0.23, 2.92]
Burnett 1999 0.87 [0.41, 1.82]
Law-Min 2003 0.92 [0.53, 1.61]
Gajwani 2016.1 1.14 [0.73, 1.78]
Agius 2008 1.26 [0.71, 2.24]
Mann 2014.3 1.38 [0.79, 2.41]
Ajnakina 2017.2 1.57 [0.82, 2.97]
Rotenberg 2017.1 1.74 [1.05, 2.90]
Bowers 2009.2 2.60 [2.07, 3.27]
Bebbington 1994 2.63 [1.35, 5.11]
Johnson 1998.1 2.79 [1.40, 5.56]
Morgan 2005.2 2.88 [1.84, 4.51]
Parkman 1997 3.14 [1.56, 6.34]
Callan 1996 3.17 [2.04, 4.92]
Thornicroft 1999.1 3.22 [2.01, 5.16]
Singh 1998 3.29 [1.71, 6.33]
Mohan 2006 3.58 [1.71, 7.52]
Ineichen 1984 4.34 [2.39, 7.88]
Moodley 1991 4.58 [2.49, 8.40]
Reeves 2002 4.66 [2.09, 10.38]
Lawlor 2012.3 4.90 [1.96, 12.24]
Thomas 1993 5.52 [2.52, 12.06]
Crowley 1992 6.28 [2.49, 15.86]
Owens 1991 7.67 [3.58, 16.44]
RE Model for compulsory detention in Black Caribbean ethnic groups ($I^2$ = 70.69%) 2.53 [2.02, 3.16]
Black unspecified minority groups
Janku 2009 0.56 [0.29, 1.10]
Coid 2000 0.73 [0.58, 0.92]
Hamilton 2015 0.85 [0.75, 0.97]
Archie 2010 0.88 [0.24, 3.15]
Goater 1999 1.13 [0.65, 1.97]
Sohler 2004 1.31 [0.82, 2.09]
Singh 2014 1.35 [1.14, 1.60]
Cole 1995 1.71 [0.95, 3.10]
Gray Houston 2001 1.81 [1.02, 3.22]
Mann 2014.2 1.91 [1.02, 3.56]
Borschmann 2010 2.21 [1.24, 3.95]
Lloyd 1992 2.22 [1.48, 3.33]
Corrigal 2013 2.89 [1.56, 5.35]
Bhui 1998.1 3.34 [0.59, 18.86]
Tolmac 2004 3.47 [1.87, 6.46]
Koffman 1997 3.95 [2.19, 7.11]
Weich 2017 4.35 [4.21, 4.49]
Singh 2015 4.67 [1.77, 12.32]
Lawlor 2012.2 5.43 [2.26, 13.05]
Commander 1999 5.48 [2.99, 10.04]
RE Model for compulsory detention in Black unspecified ethnic groups ($I^2$ = 98.08%) 2.00 [1.28, 3.11]
0
1
2
3
4
5
6
7
8
Odds Ratio

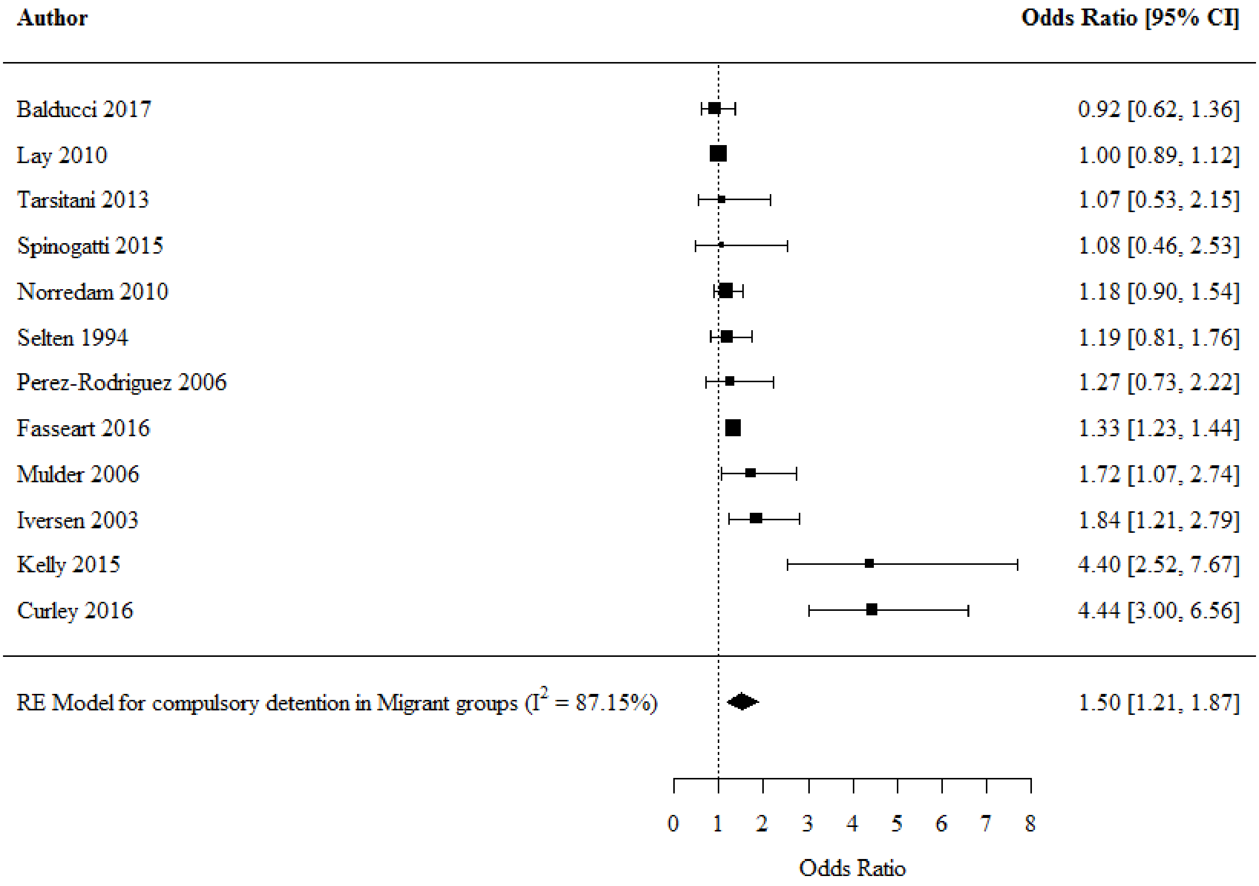
Author
Odds Ratio [95% CI]
Balducci 2017 0.92 [0.62, 1.36]
Lay 2010 1.00 [0.89, 1.12]
Tarsitani 2013 1.07 [0.53, 2.15]
Spinogatti 2015 1.08 [0.46, 2.53]
Norredam 2010 1.18 [0.90, 1.54]
Selten 1994 1.19 [0.81, 1.76]
Perez-Rodriguez 2006 1.27 [0.73, 2.22]
Fasseart 2016 1.33 [1.23, 1.44]
Mulder 2006 1.72 [1.07, 2.74]
Iversen 2003 1.84 [1.21, 2.79]
Kelly 2015 4.40 [2.52, 7.67]
Curley 2016 4.44 [3.00, 6.56]
RE Model for compulsory detention in Migrant groups ($I^2$ = 87.15%) 1.50 [1.21, 1.87]
0 1 2 3 4 5 6 7 8
Odds Ratio

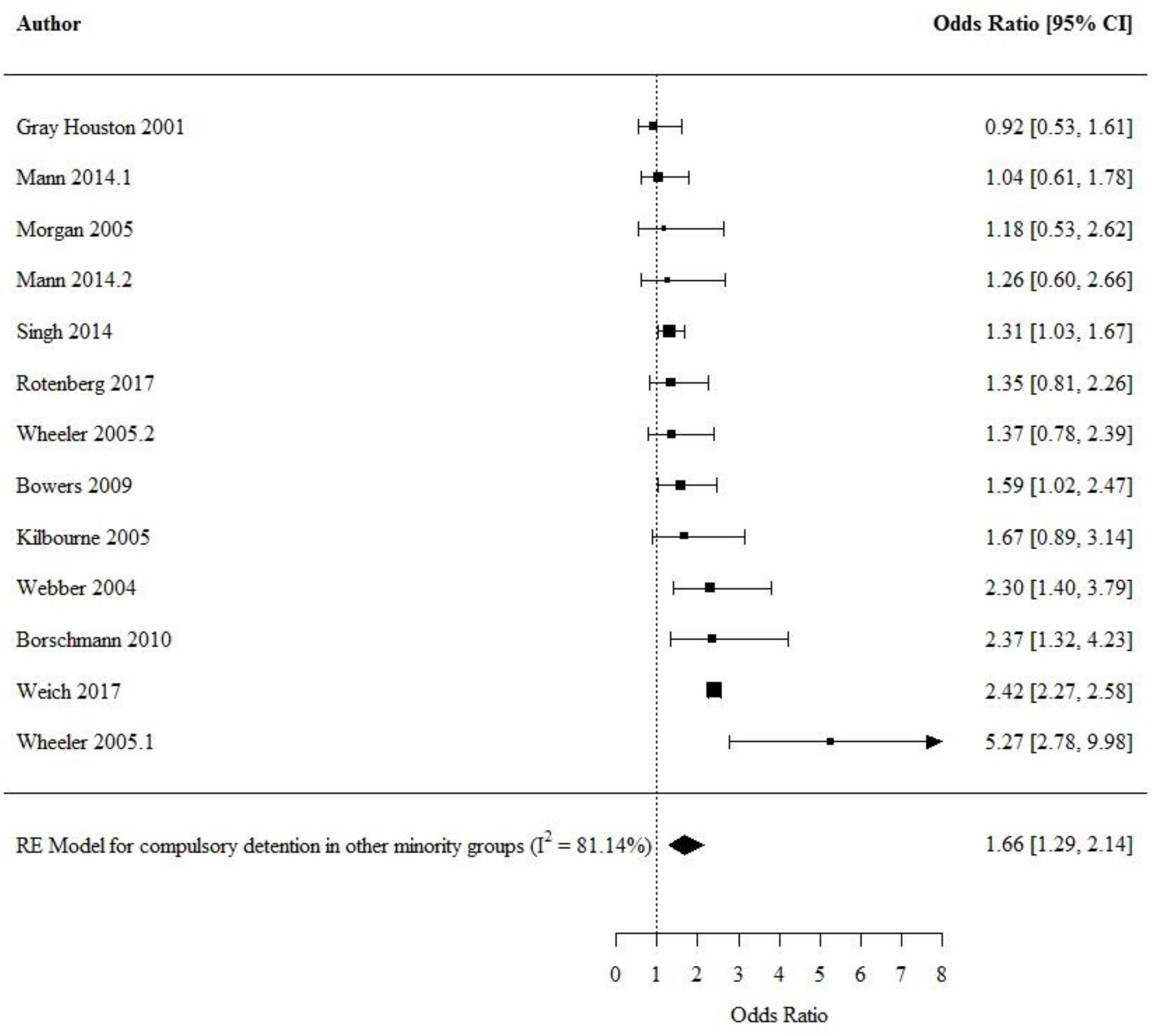
Author
Odds Ratio [95% CI]
Gray Houston 2001
0.92 [0.53, 1.61]
Mann 2014.1
1.04 [0.61, 1.78]
Morgan 2005
1.18 [0.53, 2.62]
Mann 2014.2
1.26 [0.60, 2.66]
Singh 2014
1.31 [1.03, 1.67]
Rotenberg 2017
1.35 [0.81, 2.26]
Wheeler 2005.2
1.37 [0.78, 2.39]
Bowers 2009
1.59 [1.02, 2.47]
Kilbourne 2005
1.67 [0.89, 3.14]
Webber 2004
2.30 [1.40, 3.79]
Borschmann 2010
2.37 [1.32, 4.23]
Weich 2017
2.42 [2.27, 2.58]
Wheeler 2005.1
5.27 [2.78, 9.98]
RE Model for compulsory detention in other minority groups ($I^2$ = 81.14%)
1.66 [1.29, 2.14]
0
1
2
3
4
5
6
7
8
Odds Ratio

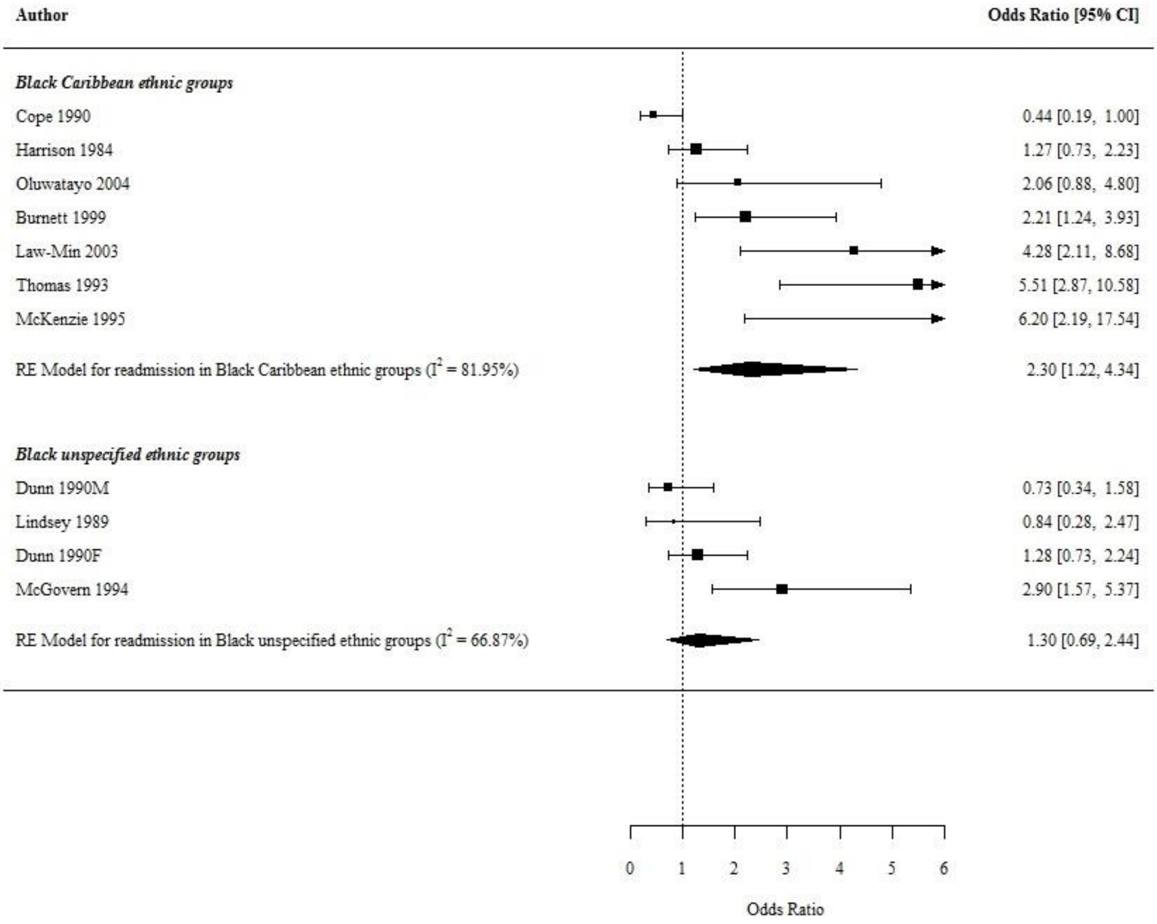

Author
Odds Ratio [95% CI]
Black Caribbean ethnic groups
Cope 1990
0.44 [0.19, 1.00]
Harrison 1984
1.27 [0.73, 2.23]
Oluwatayo 2004
2.06 [0.88, 4.80]
Burnett 1999
2.21 [1.24, 3.93]
Law-Min 2003
4.28 [2.11, 8.68]
Thomas 1993
5.51 [2.87, 10.58]
McKenzie 1995
6.20 [2.19, 17.54]
RE Model for readmission in Black Caribbean ethnic groups (I² = 81.95%)
2.30 [1.22, 4.34]
Black unspecified ethnic groups
Dunn 1990M
0.73 [0.34, 1.58]
Lindsey 1989
0.84 [0.28, 2.47]
Dunn 1990F
1.28 [0.73, 2.24]
McGovern 1994
2.90 [1.57, 5.37]
RE Model for readmission in Black unspecified ethnic groups (I² = 66.87%)
1.30 [0.69, 2.44]
0
1
2
3
4
5
6
Odds Ratio

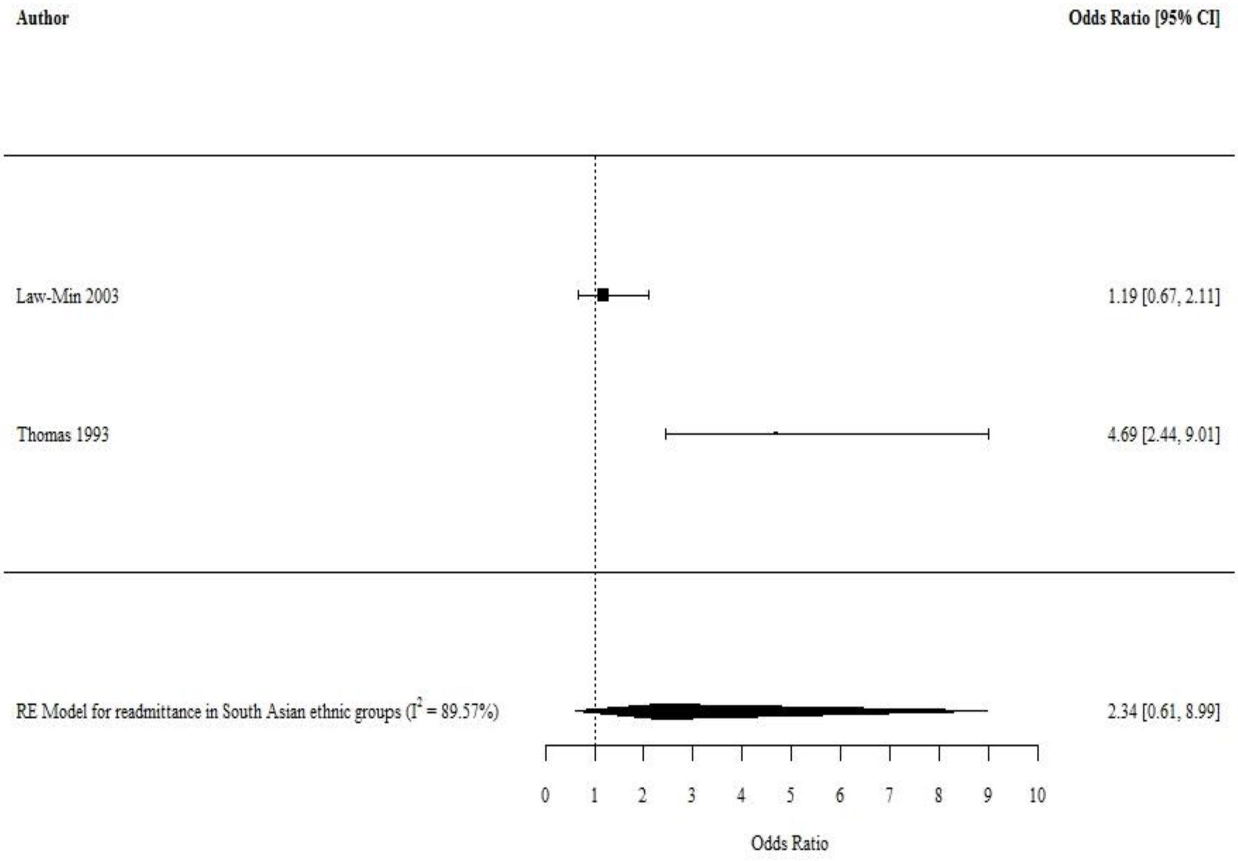
Author
Odds Ratio [95% CI]
Law-Min 2003
1.19 [0.67, 2.11]
Thomas 1993
4.69 [2.44, 9.01]
RE Model for readmittance in South Asian ethnic groups (I^2 = 89.57%)
2.34 [0.61, 8.99]
0
1
2
3
4
5
6
7
8
9
10
Odds Ratio

**Scatter plots for metaregression analyses**

**Black unspecified**

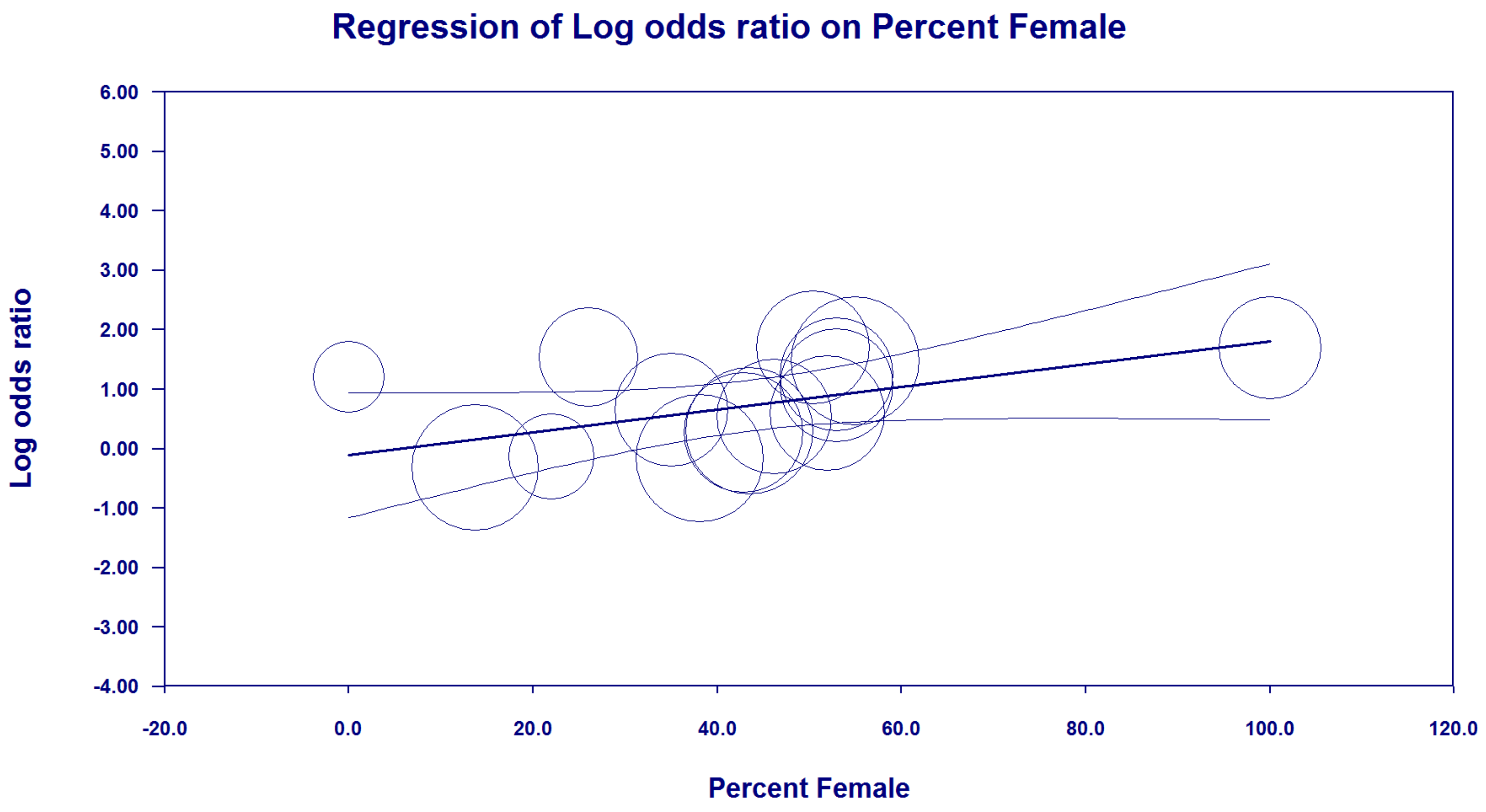

**Black Unspecified**

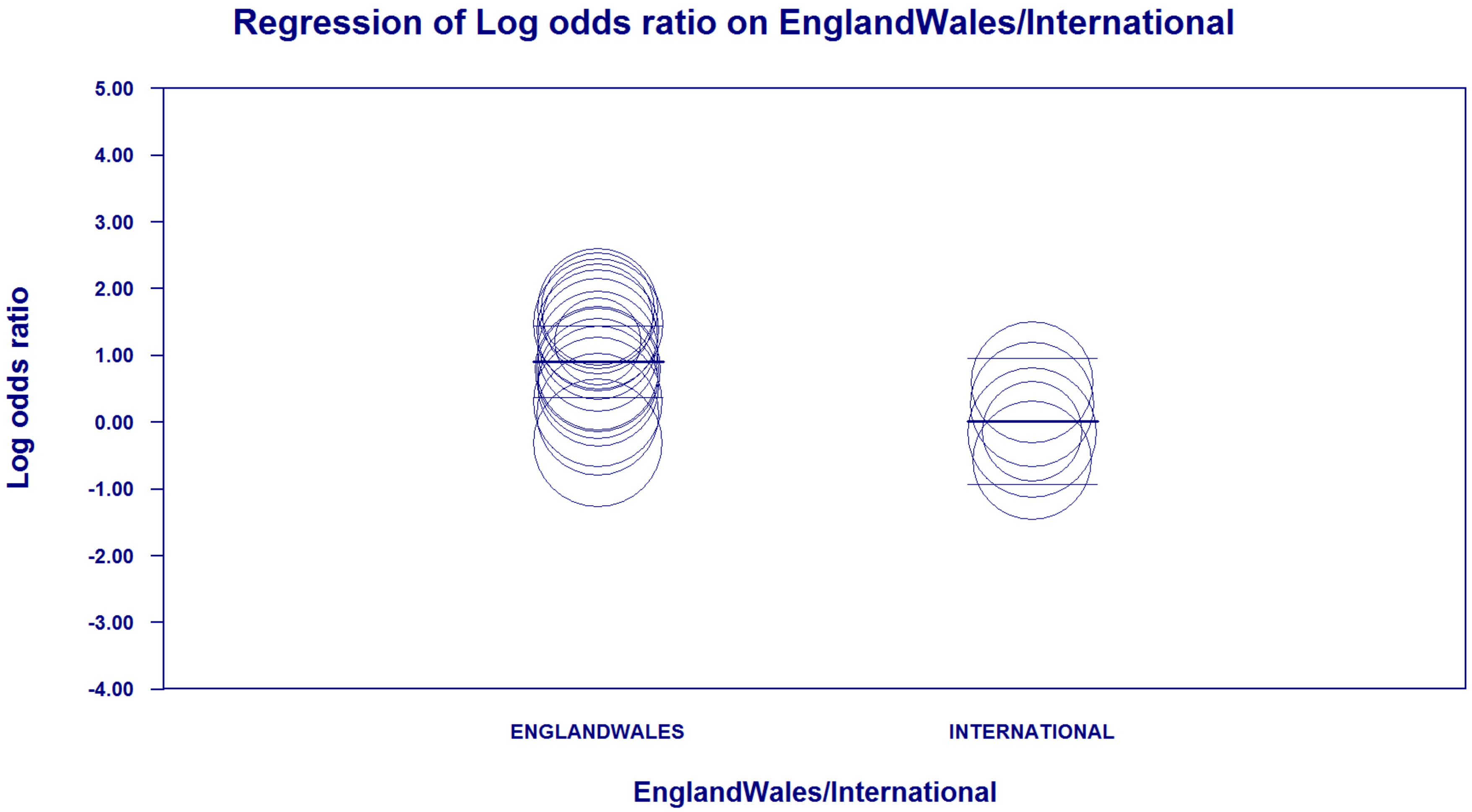

**Black Caribbean**

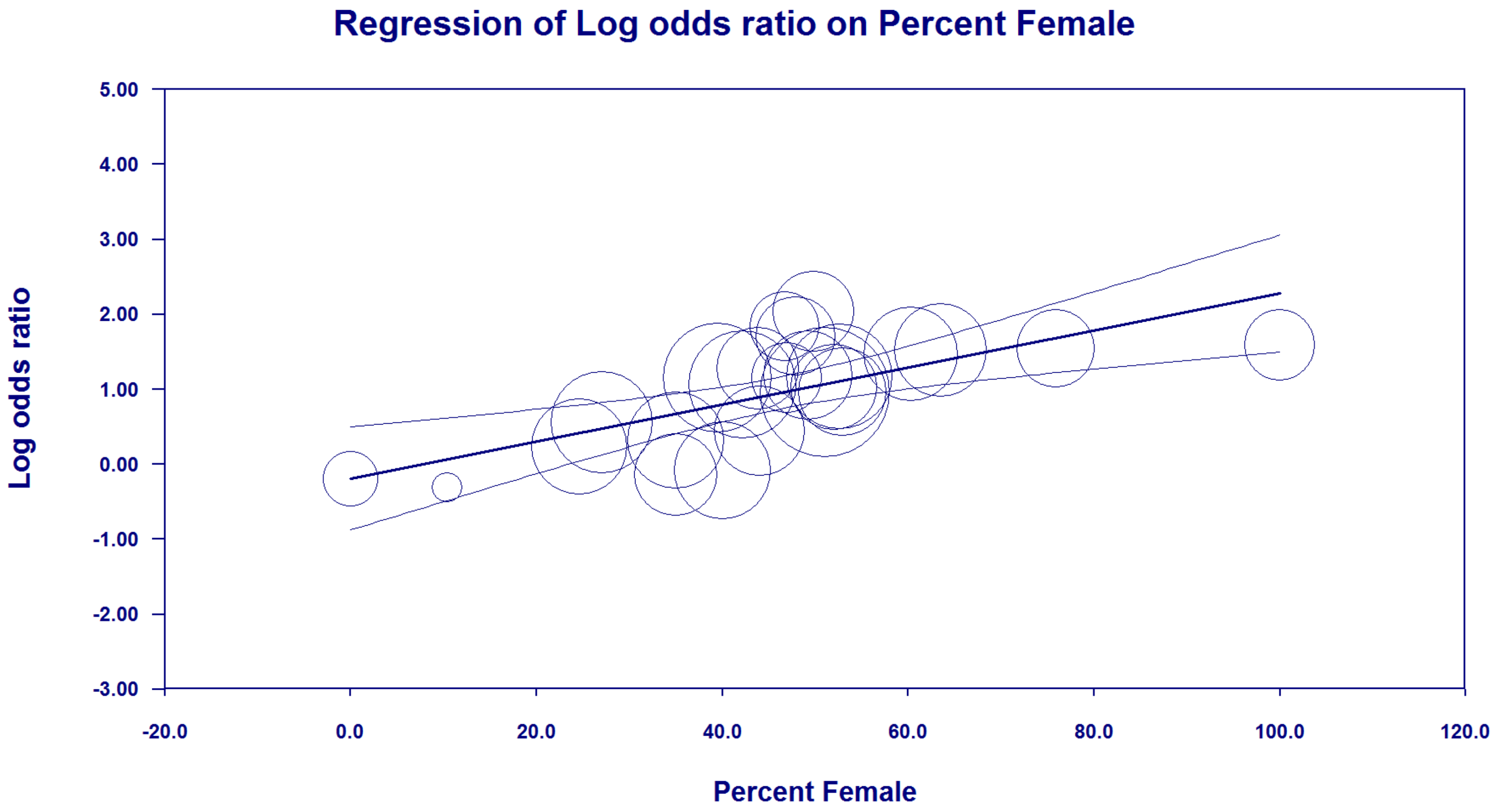

**Black Caribbean**

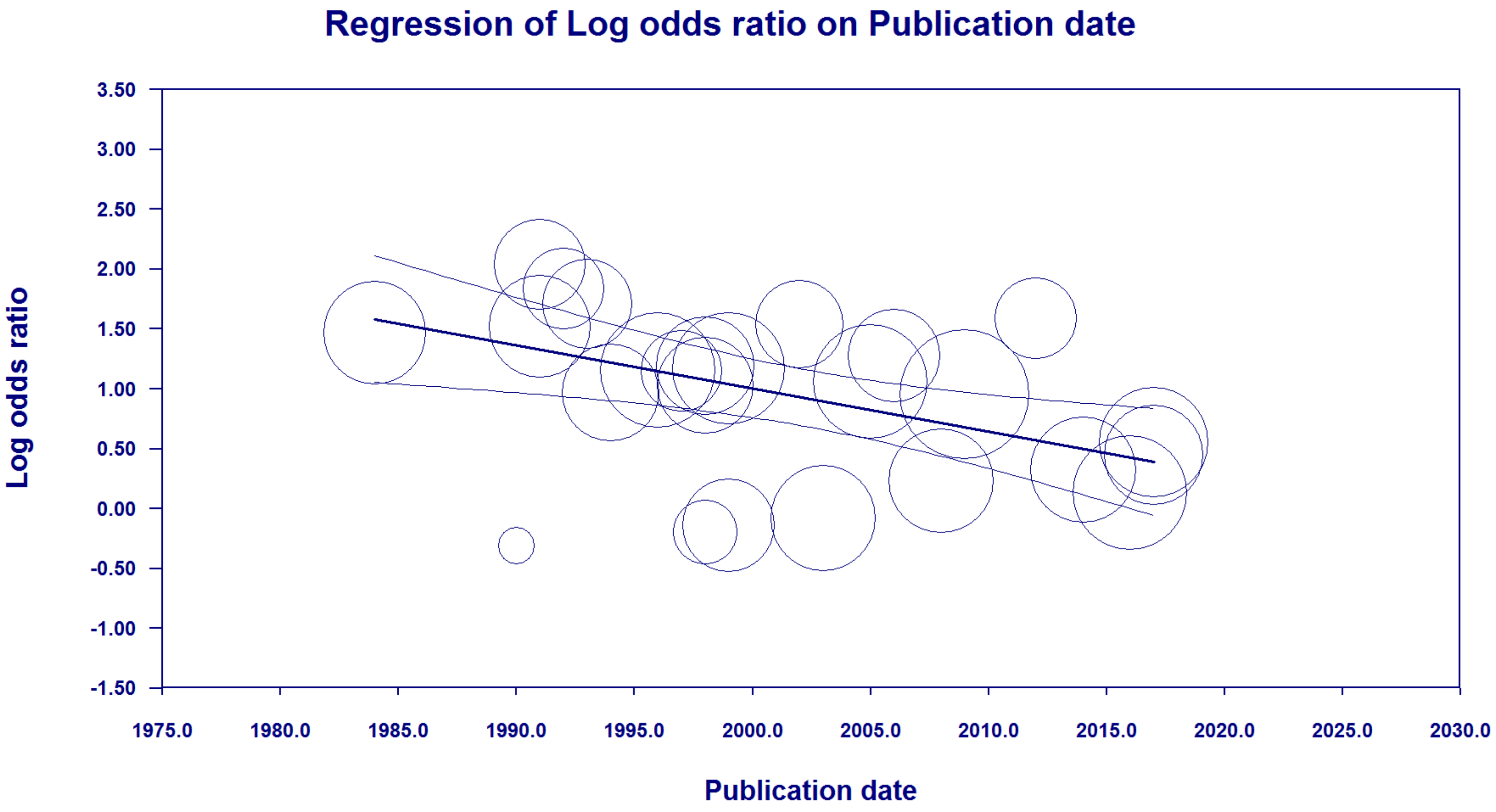

**South Asian**

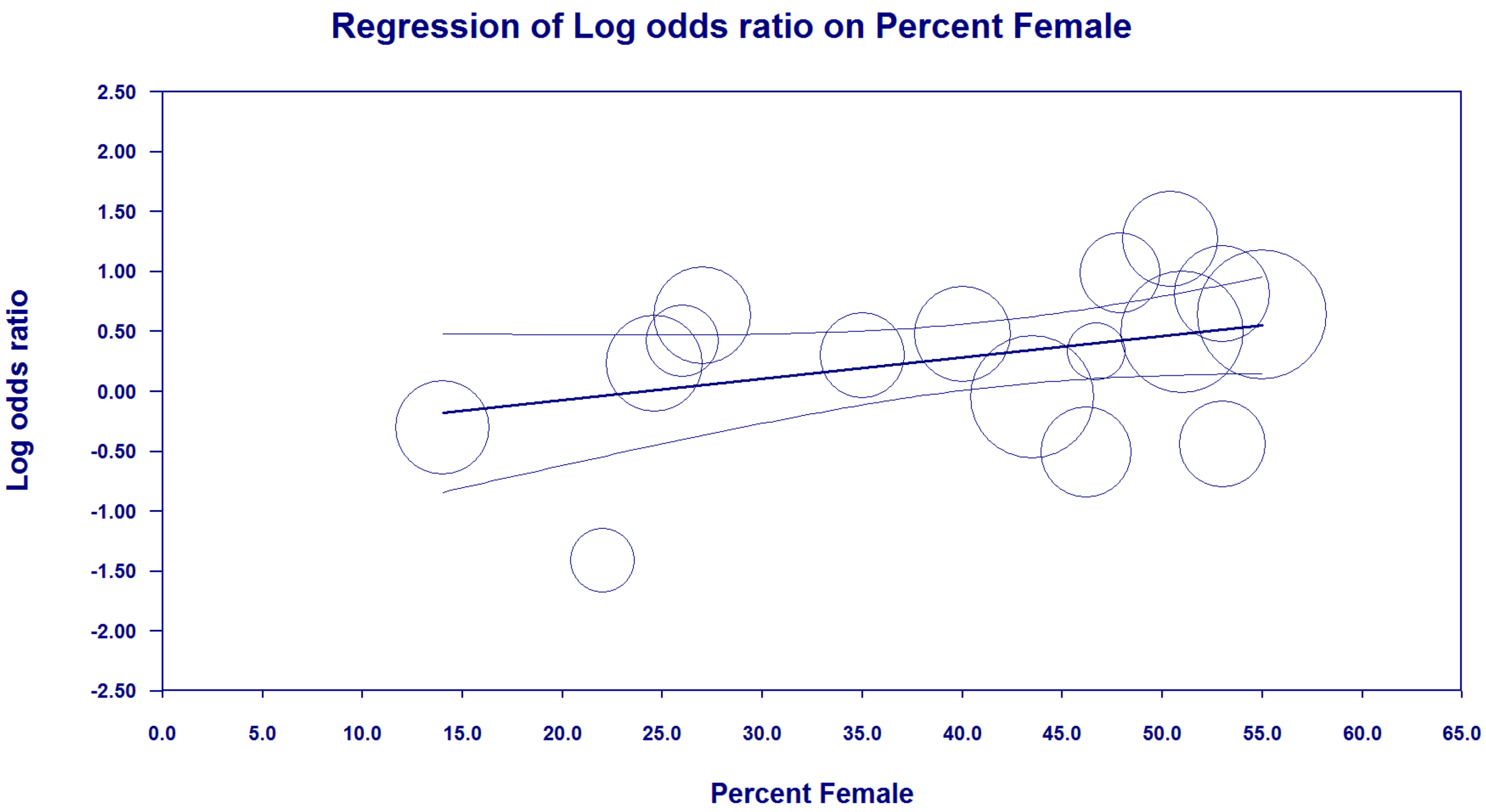

Regression of Log odds ratio on Percent Female
2.50
2.00
1.50
1.00
0.50
0.00
-0.50
-1.00
-1.50
-2.00
-2.50
Log odds ratio
0.0
5.0
10.0
15.0
20.0
25.0
30.0
35.0
40.0
45.0
50.0
55.0
60.0
65.0
Percent Female

**Sensitivity Analysis-inclusion of 4 studies reporting only adjusted data.** [32, 34, 35]

| Ethnic Group | Original Effect Size | 95% CI | K | p-value | $I^2$ | New Effect Size | 95% CI | K | p-value | $I^2$ |
|---|---|---|---|---|---|---|---|---|---|---|
| *Black groups* | | | | | | | | | | |
| Black African | 2·27 | 1·62-3·19 | 10 | <·0001 | 71·11% | 2·22 | 1·60-3·08 | 11 | <·0001 | 68.62% |
| Black Caribbean | 2·53 | 2·03-3·16 | 25 | <·0001 | 70·69% | 2·46 | 1·98-3·06 | 27 | <·0001 | 69.19% |
| Black, Unspecified | 2·00 | 1·28-3·11 | 20 | 0·0022 | 98·08% | 2·01 | 1·31-3·09 | 21 | 0·0015 | 97.98% |
| *Asian Groups* | | | | | | | | | | |
| South Asian | 1·33 | 1·07-1·65 | 20 | 0·0091 | 83·38% | No additional studies | | | | |
| East Asian | 2·17 | 1·47-3·22 | 3 | 0·0001 | 8·88% | No additional studies | | | | |
| *Amalgamated Groups* | | | | | | | | | | |
| Other minority ethnicities | 1·66 | 1·29-2·14 | 13 | <·0001 | 81·14% | No additional studies | | | | |
| Migrants | 1·50 | 1·21-1·87 | 12 | 0·0003 | 87·15% | 1·60 | 1·18-2·16 | 13 | 0·0026 | 97.45% |

Note. One study [33] reporting only adjusted data provided only Adjusted Risk Ratios and adjusted for multiple variables in addition to the demographic data adjusted for by the other studies, and so could not be combined in sensitivity analyses. This study adjusted for Age, Sex, car ownership and housing tenure.

**Explanations of psychiatric detention in Black, Asian and Minority Ethnic groups, and level of supporting evidence**

| Evidence Base | Patient related explanations | | | Illness-related explanations | | Service-related explanations | | Culture-related explanations | | | Service Patient interface | |
|---|---|---|---|---|---|---|---|---|---|---|---|---|
| *No supporting primary evidence* | Higher rates of comorbid drugs in BAME | Language barriers | Socioeconomic status | Illness expressed as violence/challenge | - | Poorer detection, diagnosis and referral for mental illness | - | Different explanatory models of illness | Greater stigma in BAME | Public social life of BAME | Cultural incompetence of services | Poor service engagement |
| *Mix of supportive and contradictory primary evidence* | BAME perceived greater risk violence/disturbance | Delayed health seeking/ poor social support | Higher prevalence/ diagnosis of psychoses in BAME | Denial of illness (poor insight) | - | BAME larger police contact | - | Ethnic disadvantage, societal racism | - | - | Absence of/ unwillingness to consult GP | - |
| *Supported by primary evidence* | - | - | - | Poor adherence | Residential instabilty | Racial bias in patient treatment | Site of service related effects | BAME perceptions of mental illness and services | Ethnic density influencing utilisation | - | Alienation from services/ mistrust | - |

## Papers with no explanations

| Title | Year |
|---|---|
| Agius | 2008 |
| Ajnakina | 2017 |
| Archie | 2010 |
| Borschmann | 2010 |
| Gray Houston | 2001 |
| Hamilton | 2015 |
| Iversen | 2003 |
| Johnson | 1998 |
| Lay | 2010 |
| Perez-Rodriguez | 2006 |
| Priebe | 2009 |
| Wheeler | 2005 |

**Papers with only unsupported explanations, by domain**

| Domain | Author | Year | Explanations |
| --- | --- | --- | --- |
| ***Patient related explanations*** | **Cope** | **1990** | BAME perceived greater risk violence/disturbance |
| | **Crowley** | **1992** | Language barriers |
| | **Curley** | **2016** | Delayed health seeking/ poor social support |
| | **Law-Min** | **2003** | Higher rates of comorbid drugs in BAME |
| | **-** | **-** | Delayed health seeking/ poor social support |
| | **-** | **-** | Delayed health seeking/ poor social support |
| | **Lay** | **2005** | Higher prevalence/ diagnosis of psychoses in BAME |
| | **Mann** | **2014** | Higher prevalence/ diagnosis of psychoses in BAME |
| | **Noredam** | **2010** | Higher prevalence/ diagnosis of psychoses in BAME |
| | **-** | **-** | Language barriers |
| | **Owens** | **1991** | Delayed health seeking/ poor social support |
| | **Reeves** | **2002** | Delayed health seeking/ poor social support |
| | **Singh** | **1998** | BAME perceived greater risk violence/disturbance |
| | **-** | **-** | Delayed health seeking/ poor social support |
| | **Tarsitani** | **2013** | Language barriers |
| ***Illness-related explanations*** | **Cole** | **1995** | Denial of illness (poor insight) |
| | **Koffman** | **1997** | Illness expressed as violence/challenge |
| | **Law-Min** | **2003** | Denial of illness (poor insight) |
| | **-** | **-** | Denial of illness (poor insight) |
| | **McGovern** | **1994** | Illness expressed as violence/challenge |
| | **Singh** | **1998** | Illness expressed as violence/challenge |
| | **-** | **-** | Poor adherence |
| | **-** | **-** | Denial of illness (poor insight) |
| ***Service-related explanations*** | **Cope** | **1990** | BAME larger police contact |
| | **Crowley** | **1992** | Poorer detection, diagnosis and referral for mental illness |
| | **-** | **-** | Racial bias in patient treatment |
| | **Law-Min** | **2003** | Poorer detection, diagnosis and referral for mental illness |
| | **-** | **-** | Poorer detection, diagnosis and referral for mental illness |

| | | | |
|---|---|---|---|
| | **-** | **-** | Poorer detection, diagnosis and referral for mental illness |
| | **Mann** | **2014** | Racial bias in patient treatment |
| | **McGovern** | **1994** | Poorer detection, diagnosis and referral for mental illness |
| | **-** | **-** | BAME larger police contact |
| | **-** | **-** | Racial bias in patient treatment |
| | **-** | **-** | Racial bias in patient treatment |
| | **-** | **-** | Racial bias in patient treatment |
| | **Noredam** | **2010** | Poorer detection, diagnosis and referral for mental illness |
| | **Takei** | **1998** | Poorer detection, diagnosis and referral for mental illness |
| | **-** | **-** | Racial bias in patient treatment |
| | **-** | **-** | Racial bias in patient treatment |
| | **Tolmac** | **2004** | BAME larger police contact |
| ***Culture-related explanations*** | **Balducci** | **2017** | Ethnic disadvantage, societal racism |
| | **Cole** | **1995** | Greater stigma in BAME |
| | **Kelly** | **2015** | Different explanatory models of illness |
| | **Koffman** | **1997** | Public social life of BAME |
| | **Law-Min** | **2003** | Greater stigma in BAME |
| | **Lay** | **2005** | Different explanatory models of illness |
| | **McGovern** | **1994** | Ethnic disadvantage, societal racism |
| | **Noredam** | **2010** | Greater stigma in BAME |
| | **-** | **-** | Ethnic disadvantage, societal racism |
| | **Spinogatti** | **2015** | Ethnic disadvantage, societal racism |
| | **-** | **-** | Ethnic density influencing utilisation |
| ***Service Patient interface*** | **Ali** | **2007** | Cultural incompetence of services |
| | **Balducci** | **2017** | Poor service engagement |
| | **Cole** | **1995** | Alienation from services/ mistrust |
| | **Cope** | **1990** | Alienation from services/ mistrust |
| | **Crowley** | **1992** | Alienation from services/ mistrust |
| | **-** | **-** | Cultural incompetence of services |
| | **Davies** | **1996** | Alienation from services/ mistrust |

| | | |
|---|---|---|
| **-** | **-** | Alienation from services/ mistrust |
| **Ineichen** | **1984** | Absence of/ unwillingness to consult GP |
| **Law-Min** | **2003** | Alienation from services/ mistrust |
| **-** | **-** | Absence of/ unwillingness to consult GP |
| **Mann** | **2014** | Absence of/ unwillingness to consult GP |
| **Noredam** | **2010** | Alienation from services/ mistrust |
| **-** | **-** | Cultural incompetence of services |
| **Singh** | **1998** | Poor service engagement |
| **Spinogatti** | **2015** | Alienation from services/ mistrust |
| **-** | **-** | Poor service engagement |
| **Takei** | **1998** | Alienation from services/ mistrust |

## Papers with at least one explanation supported by primary evidence, by domain

| Title | Year | Explanations | Primary Evidence | Note |
|---|---|---|---|---|
| | | **Patient-related** | | |
| **Afuwape** | 2006 | Higher rates of comorbid drugs in BAME | Yes - against | Comorbid substance use was not an independent risk factor contributing to compulsory admission in black individuals |
| **Bebbington** | 1994 | Higher prevalence/ diagnosis of psychoses in BAME | Yes | In the first model of high rates of compulsory admissions among Black-Caribbeans, the effect could be explained by the interation of diagnosis and challenging behaviour. In a second model "a small proportion of [of the excess] could reflect increased readiness to compulsory admit Black Caribbeans. |
| **-** | - | BAME perceived greater risk violence/disturbance | Yes - against | No interaction between ethnicity and challenging behaviour |
| **Bhui** | 1998 | Higher prevalence/ diagnosis of psychoses in BAME | Yes | Receiving mental health act is more associated with diagnosis than ethnicity |
| **Bowers** | 2009 | Higher prevalence/ diagnosis of psychoses in BAME | Yes - against | Association of ethnicity and detentention remains after adjusting for age, gender, diagnosis and risk |
| **Callan** | 1996 | Higher prevalence/ diagnosis of psychoses in BAME | Yes - against | Association of ethnicity and detentention remains after adjusting for age, gender, diagnosis and risk |
| **Coid** | 2000 | Socioeconomic status | Yes - against | Both african caribbean and asian patients come from socioeconomically deprived areas but african caribbean patients more likely to be detained |
| **Commander** | 1999 | BAME perceived greater risk violence/disturbance | Yes | An association |
| **-** | - | Delayed health seeking/ poor social support | Yes | An association due to differences in social support |
| **de Wit** | 2012 | BAME perceived greater risk violence/disturbance | Yes | Most patients who were described as a danger to others were men from the major migrant groups, even after adjustment for the type of disorder, with the highest risk for second generation Turkish migrants |
| **Dunn** | 1990 | Higher prevalence/ diagnosis of psychoses in BAME | Yes | An association |
| **Goater** | 1999 | Higher prevalence/ diagnosis of psychoses in BAME | Yes | An association |
| **Harrison** | 1984 | BAME perceived greater risk violence/disturbance | Yes - against | West Indian patients were more likely to be female, employed, socially supported and to comply and adhere to treatment |
| **-** | - | BAME perceived greater risk violence/disturbance | Yes | West Indian patients more likely to present with 'public disturbance' |
| **Kilbourne** | 2005 | Higher rates of comorbid drugs in BAME | Yes - against | Involuntary care remained associated with ethnic minority status after controlling for substance use |
| **Lawlor** | 2012 | BAME perceived greater risk violence/disturbance | Yes | Clinicians reported white-other and black women were more likely to be admitted because of an assault on an unrelated person or destruction of property |
| **Lindsey** | 1989 | BAME perceived greater risk violence/disturbance | Yes | No difference in TSBC Hostile-Belligerence Index between black voluntary and involuntary patients, but black voluntary patients higher than white voluntary |
| **Lloyd** | 1992 | BAME perceived greater risk violence/disturbance | Yes | An association |

| | | | | |
|---|---|---|---|---|
| **-** | - | BAME perceived greater risk violence/disturbance | Yes - against | Disturbed behaviour does not account for assoication between ethnicity and compulsory detention |
| **Moodley** | 1991 | Higher prevalence/ diagnosis of psychoses in BAME | Yes - against | Higher rates of psychosis do not explain higher detention tares |
| **Morgan** | 2005 | BAME perceived greater risk violence/disturbance | Yes - against | Higher detention not explained by perceived risk |
| **-** | - | Delayed health seeking/ poor social support | Yes - against | Social isolation does not account for ethnic differences in compulsory admission |
| **Mulder** | 2006 | BAME perceived greater risk violence/disturbance | Yes | Association between non-western ethnicity and compulsory admission found to be explained by a greater severity of psychiatric symptoms, greater level of threat, more lack of treatment motivation and lower level of functioning |
| **Thomas** | 1993 | Delayed health seeking/ poor social support | Yes | An association for african-caribbean but not asian patients |
| **Webber** | 2004 | Delayed health seeking/ poor social support | Yes | Lower social support associated with s.4 admission |
| | | **Illness-related explanations** | | |
| **Commander** | 1999 | Denial of illness (poor insight) | Yes | An association |
| **McKenzie** | 1995 | Illness expressed as violence/challenge | Yes - against | Confirms no different of illness/clinical state |
| **Mulder** | 2006 | Denial of illness (poor insight) | Yes - against | Lack of awareness of illness was not associated with comp[ulsory admission |
| **Oluwatayo** | 2004 | Poor adherence | Yes | Compulsorily admitted african caribbean patients were more likely to comply poorly with their medications |
| **Owens** | 1991 | Illness expressed as violence/challenge | Yes - against | Confirms no difference between black and white sectioned patients in publicly manifest disturbance |
| **Rotenberg** | 2017 | residential instability | Yes | An association |
| | | **Service-related explanations** | | |
| **Afuwape** | 2006 | BAME larger police contact | Yes - against | No signifcant differences between groups in rates of police contact |
| **Corrigal** | 2013 | Racial bias in patient treatment | Yes | Outsize cumpolsory detention for black patients with psychotic, but not other, dianoses - possibility that clinicians are showing bias in interpretation of risk associated with psychotic presentation in black and "other" ethnic groups |
| **Fassaert** | 2016 | BAME larger police contact | Yes | Ethnic minorities more likely to be referred through police |
| **Gajwani** | 2016 | BAME larger police contact | Yes | The rate of criminal justice involvement was greater amongst the Black African Group |
| **Lawlor** | 2012 | BAME larger police contact | Yes - non significant | Rates of police and criminal justice system contact in the pathway to care for the black caribbean group were raised, but not significantly so |
| **Morgan** | 2005 | BAME larger police contact | Yes - against | Criminal justice involvement did not fully account for ethnic variation in detention |
| **Selten** | 1994 | Socio-economic status | Yes-against | Found little support that socio-economic deprivation was an important facor |
| **Singh** | 2014 | Site of service related effects | Yes | Site of service accounts for ethnic differences |
| **Sohler** | 2004 | BAME larger police contact | Yes | Greater involvement of police/EMS in admission despite no differences in dangerous behaviours or substance abuse |
| **Thornicroft** | 1999 | BAME larger police contact | Yes | black african and black caribbean patients have great contact with the police and forensic services |
| | | **Culture-related explanations** | | |
| **Bansal** | 2014 | Ethnic disadvantage, societal racism | Yes - against | Adjusting for SES increased the risk of mental health hospitalisation for the pakistanki group |
| **Bowers** | 2009 | Ethnic disadvantage, societal racism | Yes | greater police detention |

| | | | | |
|---|---|---|---|---|
| **Goater** | 1999 | BAME perceptions of mental illness and services | Yes | Higher referral to crisis services from police |
| **Janku** | 2009 | Ethnic disadvantage, societal racism | Yes | decreased referral within juvenile system -african american youth face a more disadvataged environment than caucasian counterparts, mediating referral to mental health services |
| **Rotenberg** | 2017 | Ethnic density influencing utilisation | Yes | Ethnic density found to be protective |
| **Singh** | 2015 | Different explanatory models of illness | Yes - against | differences in compulsory admission not driven by illness atrribution |
| **Weich** | 2017 | Ethnic disadvantage, societal racism | Yes | statistically significant associations with area-level deprivation and ethnic density, with risk of compulsory admission inversely associated with the proportion of white British LSOA residents. |
| | | **Service Patient interface** | | |
| **Burnett** | 1999 | Absence of/ unwillingness to consult GP | Yes | An association |
| **Callan** | 1996 | Absence of/ unwillingness to consult GP | Yes | this study did not detect differences in rates of GP referrals for the index admission, but for subsequent admissions a lower proportion of Afro-Caribbean patients were referred by their GP. |
| **Commander** | 1999 | Alienation from services/ mistrust | Yes | An association |
| **Fassaert** | 2016 | Absence of/ unwillingness to consult GP | Yes | non-ethnic dutch were less liely to access specialised mental health care through primary care/GPs |
| **Mohan** | 2006 | Absence of/ unwillingness to consult GP | Yes - against | african-caribbean patients in this study had relatively more contact with GPs in the period before intensive community treatment was introduced, contrasting with previous research |
| **Oluwatayo** | 2004 | Absence of/ unwillingness to consult GP | Yes | compulsorily admitted african caribbean patients were more likely to not contact their GP |
| **Parkman** | 1997 | Alienation from services/ mistrust | Yes | second generation black caribbean patients have decreasing satisfaction with each hospital admission |

**References included in the review**


1. Afuwape SA, Johnson S, Craig TJK, Miles H, Leese M, Mohan R, et al. Ethnic differences among a community cohort of individuals with dual diagnosis in South London. J Ment Health. 2006;15(5):551-67.
2. Agius M, Shah S, Ramkisson R, Persaud A, Murphy S, Zaman R. Three year outcomes in an early intervention service for psychosis in a multicultural and multiethnic population. Psychiatr. 2008;20(4):494-9.
3. Ajnakina O, Lally J, Di Forti M, Kolliakou A, Gardner-Sood P, Lopez-Morinigo J, et al. Patterns of illness and care over the 5 years following onset of psychosis in different ethnic groups; the GAP-5 study. Soc Psychiatry Psychiatr Epidemiol. 2017;05:05.
4. Ali S, Dearman SP, McWilliam C. Are Asians at greater risk of compulsory psychiatric admission than Caucasians in the acute general adult setting? Med Sci Law. 2007;47(4):311-4.
5. Archie S, Akhtar-Danesh N, Norman R, Malla A, Roy P, Zipursky RB. Ethnic diversity and pathways to care for a first episode of psychosis in Ontario. Schizophr Bull. 2010;36(4):688-701.
6. Balducci PM, Bernardini F, Pauselli L, Tortorella A, T Compton M. Correlates of involuntary admission: findings from an Italian inpatient psychiatric unit. Psychiatr. 2017;29(4):490-6.
7. Bansal N, Bhopal R, Netto G, Lyons D, Steiner MF, Sashidharan SP. Disparate patterns of hospitalisation reflect unmet needs and persistent ethnic inequalities in mental health care: the Scottish health and ethnicity linkage study. Ethn Health. 2014;19(2):217-39.
8. Bebbington PE, Feeney ST, Flannigan CB, Glover GR, Lewis SW, Wing JK. Inner london collaborative audit of admissions in two health districts II: Ethnicity and the use of the mental health act. Br J Psychiatry. 1994;165(DEC.):743-9.
9. Bhui K, Brown P, Hardie T, Watson JP, Parrott J. African-Caribbean men remanded to Brixton Prison. Psychiatric and forensic characteristics and outcome of final court appearance. Br J Psychiatry. 1998;172(APR.):337-44.
10. Borschmann RD, Gillard S, Turner K, Lovell K, Goodrich-Purnell N, Chambers M. Demographic and referral patterns of people detained under Section 136 of the Mental Health Act (1983) in a south London Mental Health Trust from 2005 to 2008. Med Sci Law. 2010;50(1):15-8.
11. Bowers L, Jones J, Simpson A. The demography of nurses and patients on acute psychiatric wards in England. J Clin Nurs. 2009;18(6):884-92.
12. Burnett R, Mallett R, Bhugra D, Hutchinson G, Der G, Leff J. The first contact of patients with schizophrenia with psychiatric services: Social factors and pathways to care in a multi-ethnic population. Psychol Med. 1999;29(2):475-83.
13. Callan AF. Schizophrenia in Afro-Caribbean immigrants. J R Soc Med. 1996;89(5):253-6.
14. Coid J, Kahtan N, Gault S, Jarman B. Ethnic differences in admissions to secure forensic psychiatry services. Br J Psychiatry. 2000;177(SEPT):241-7.
15. Cole E, Leavey G, King M, Johnson-Sabine E, Hoar A. Pathways to care for patients with a first episode of psychosis: A comparison of ethnic groups. Br J Psychiatry. 1995;167(DEC.):770-6.
16. Commander MJ, Cochrane R, Sashidharan SP, Akilu F, Wildsmith E. Mental health care for Asian, black and white patients with non-affective psychoses: Pathways to the psychiatric hospital, in-patient and after-care. Social Psychiatry and Psychiatric Epidemiology. 1999;34(9):484-91.
17. Cope R, Ndegwa D. Ethnic differences in admission to a regional secure unit. The Journal of Forensic Psychiatry. 1990;1(3):365-78.
18. Corrigall R, Bhugra D. The role of ethnicity and diagnosis in Rates of adolescent psychiatric admission and compulsory detention: A longitudinal case-note study. J R Soc Med. 2013;106(5):190-5.
19. Crowley JJ, Simmons S. Mental health, race and ethnicity: a retrospective study of the care of ethnic minorities and whites in a psychiatric unit. J Adv Nurs. 1992;17(9):1078-87.
20. Curley A, Agada E, Emechebe A, Anamdi C, Ng XT, Duffy R, et al. Exploring and explaining involuntary care: the relationship between psychiatric admission status, gender and other demographic and clinical variables. International journal of law and psychiatry. 2016;47:53-9.
21. Davies S, Thornicroft G, Leese M, Higgingbotham A, Phelan M. Ethnic differences in risk of compulsory psychiatric admission among representative cases of psychosis in London. Br Med J. 1996;312(7030):533-7.
22. de Wit MA, Tuinebreijer WC, van Brussel GH, Selten JP. Ethnic differences in risk of acute compulsory admission in Amsterdam, 1996-2005. Soc Psychiatry Psychiatr Epidemiol. 2012;47(1):111-8.
23. Dunn J, Fahy TA. Police admissions to a psychiatric hospital. Demographic and clinical differences between ethnic groups. Br J Psychiatry. 1990;156(MAR.):373-8.
24. Fassaert T, Heijnen H, de Wit MA, Peen J, Beekman AT, Dekker J. The association between ethnic background and characteristics of first mental health treatment for psychotic disorders in the Netherlands between 2001 and 2005. Soc Psychiatry Psychiatr Epidemiol. 2016;51(12):1591-601.

25. Gajwani R, Parsons H, Birchwood M, Singh SP. Ethnicity and detention: are Black and minority ethnic (BME) groups disproportionately detained under the Mental Health Act 2007? Soc Psychiatry Psychiatr Epidemiol. 2016;51(5):703-11.
26. Goater N, King M, Cole E, Leavey G, Johnson-Sabine E, Blizard R, et al. Ethnicity and outcome of psychosis. Br J Psychiatry. 1999;175:34-42.
27. Gray Houston K, Mariotto M, Hays JR. Outcomes for psychiatric patients following first admission: Relationships with voluntary and involuntary treatment and ethnicity. Psychol Rep. 2001;88(3,Pt2):1012-4.
28. Hamilton JE, Heads AM, Cho RY, Lane SD, Soares JC. Racial disparities during admission to an academic psychiatric hospital in a large urban area. Compr Psychiatry. 2015;63:113-22.
29. Harrison G, Ineichen B, Smith J, Morgan HG. Psychiatric hospital admissions in Bristol: II. Social and clinical aspects of compulsory admission. The British Journal of Psychiatry. 1984;145:605-11.
30. Ineichen B, Harrison G, Morgan HG. Psychiatric hospital admissions in Bristol: I. Geographical and ethnic factors. The British Journal of Psychiatry. 1984;145:600-4.
31. Iversen VC, Morken G. Acute admissions among immigrants and asylum seekers to a psychiatric hospital in Norway. Soc Psychiatry Psychiatr Epidemiol. 2003;38(9):515-9.
32. Janku AD, Yan J. Exploring patterns of court-ordered mental health services for juvenile offenders: Is there evidence of systemic bias? Criminal Justice and Behavior. 2009;36(4):402-19.
33. Johnson S, Leese M, Brooks L, Clarkson P, Guite H, Thornicroft G, et al. Frequency and predictors of adverse events. PRiSM psychosis study 3. Br J Psychiatry. 1998;173(NOV.):376-84.
34. Kelly BD, Emechebe A, Anamdi C, Duffy R, Murphy N, Rock C. Custody, care and country of origin: Demographic and diagnostic admission statistics at an inner-city adult psychiatry unit. International Journal of Law and Psychiatry. 2015;38:1-7.
35. Kilbourne AM, Pincus H, Kirk GF, Evans D, Jaskiw G, Tekell J, et al. Clinical, psychosocial, and treatment differences in minority patients with bipolar disorder. Bipolar Disord. 2005;7(1):89-97.
36. Koffman J, Fulop NJ, Pashley D, Coleman K. Ethnicity and use of acute psychiatric beds: One-day survey in North and South Thames regions. Br J Psychiatry. 1997;171(SEPT.):238-41.
37. Lawlor C, Johnson S, Cole L, Howard LM. Ethnic variations in pathways to acute care and compulsory detention for women experiencing a mental health crisis. Int J Soc Psychiatry. 2012;58(1):3-15.
38. Law-Min R, Oyebode F, Haque MS. First compulsory admissions under part II of the Mental Health Act 1983: a retrospective five-year study. Med Sci Law. 2003;43(3):248-54.
39. Lay B, Lauber C, Rossler W. Are immigrants at a disadvantage in psychiatric in-patient care? Acta Psychiatr Scand. 2005;111(5):358-66.
40. Lay B, Nordt C, Rössler W. Variation in use of coercive measures in psychiatric hospitals. European Psychiatry. 2011;26(4):244-51.
41. Lindsey KP, Paul GL, Mariotto MJ. Urban psychiatric commitments: disability and dangerous behavior of black and white recent admissions. Hosp Community Psychiatry. 1989;40(3):286-94.
42. Lloyd K, Moodley P. Psychotropic medication and ethnicity: An inpatient survey. Social Psychiatry and Psychiatric Epidemiology. 1992;27(2):95-101.
43. Mann F, Fisher HL, Major B, Lawrence J, Tapfumaneyi A, Joyce J, et al. Ethnic variations in compulsory detention and hospital admission for psychosis across four UK Early Intervention Services. BMC Psychiatry. 2014;14 (1) (no pagination)(256).
44. McGovern D, Hemmings P, Cope R, Lowerson A. Long-term follow-up of young Afro-Caribbean Britons and white Britons with a first admission diagnosis of schizophrenia. Social Psychiatry and Psychiatric Epidemiology. 1994;29(1):8-19.
45. McKenzie K, Van Os J, Fahy T, Jones P, Harvey I, Toone B, et al. Psychosis with good prognosis in Afro-Caribbean people now living in the United Kingdom. Br Med J. 1995;311(7016):1325-8.
46. Mohan R, McCrone P, Szmukler G, Micali N, Afuwape S, Thornicroft G. Ethnic differences in mental health service use among patients with psychotic disorders. Social Psychiatry and Psychiatric Epidemiology. 2006;41(10):771-6.
47. Moodley P, Perkins RE. Routes to psychiatric inpatient care in an Inner London Borough. Social Psychiatry and Psychiatric Epidemiology. 1991;26(1):47-51.
48. Morgan C, Mallett R, Hutchinson G, Bagalkote H, Morgan K, Fearon P, et al. Pathways to care and ethnicity. 1: Sample characteristics and compulsory admission. Report from the AESOP study. Br J Psychiatry. 2005;186:281-9.
49. Mulder CL, Koopmans GT, Selten JP. Emergency psychiatry, compulsory admissions and clinical presentation among immigrants to the Netherlands. Br J Psychiatry. 2006;188(APR.):386-91.
50. Norredam M, Garcia-Lopez A, Keiding N, Krasnik A. Excess use of coercive measures in psychiatry among migrants compared with native Danes. Acta Psychiatr Scand. 2010;121(2):143-51.
51. Oluwatayo O, Gater R. The role of engagement with services in compulsory admission of African/Caribbean patients. Social Psychiatry and Psychiatric Epidemiology. 2004;39(9):739-43.

52. Owens D, Harrison G, Boot D. Ethnic factors in voluntary and compulsory admissions. Psychol Med. 1991;21(1):185-96.
53. Parkman S, Davies S, Leese M, Phelan M, Thornicroft G. Ethnic differences in satisfaction with mental health services among representative people with psychosis in South London: PRiSM study 4. Br J Psychiatry. 1997;171(SEPT.):260-4.
54. Perez-Rodriguez MM, Baca-Garcia E, Quintero-Gutierrez FJ, Gonzalez G, Saiz-Gonzalez D, Botillo C, et al. Demand for psychiatric emergency services and immigration. Findings in a Spanish hospital during the year 2003. European Journal of Public Health. 2006;16(4):383-7.
55. Priebe S, Katsakou C, Amos T, Leese M, Morriss R, Rose D, et al. Patients' views and readmissions 1 year after involuntary hospitalisation. Br J Psychiatry. 2009;194(1):49-54.
56. Reeves S, Stewart R, Howard R. Service contact and psychopathology in very-late-onset schizophrenia-life psychosis: The effects of gender and ethnicity. Int J Geriatr Psychiatry. 2002;17(5):473-9.
57. Rotenberg M, Tuck A, Ptashny R, McKenzie K. The role of ethnicity in pathways to emergency psychiatric services for clients with psychosis. BMC Psychiatry. 2017;17(1):137.
58. Selten JP, Sijben N. First admission rates for schizophrenia in immigrants to the Netherlands. Social Psychiatry and Psychiatric Epidemiology. 1994 Mar 1;29(2):71-7.
59. Singh SP, Croudace T, Beck A, Harrison G. Perceived ethnicity and the risk of compulsory admission. Social Psychiatry and Psychiatric Epidemiology. 1998;33(1):39-44.
60. Singh SP, Brown L, Winsper C, Gajwani R, Islam Z, Jasani R, et al. Ethnicity and pathways to care during first episode psychosis: The role of cultural illness attributions. BMC Psychiatry. 2015;15 (1) (no pagination)(287).
61. Singh SP, Burns T, Tyrer P, Islam Z, Parsons H, Crawford MJ. Ethnicity as a predictor of detention under the Mental Health Act. Psychol Med. 2014;44(5):997-1004.
62. Sohler NL, Bromet EJ, Lavelle J, Craig TJ, Mojtabai R. Are there racial differences in the way patients with psychotic disorders are treated at their first hospitalization? Psychol Med. 2004;34(4):705-18.
63. Spinogatti F, Civenti G, Conti V, Lora A. Ethnic differences in the utilization of mental health services in Lombardy (Italy): An epidemiological analysis. Social Psychiatry and Psychiatric Epidemiology. 2015;50(1):59-65.
64. Takei N, Persaud R, Woodruff P, Brockington I, Murray RM. First episodes of psychosis in Afro-Caribbean and White people. An 18- year follow-up population-based study. Br J Psychiatry. 1998;172(FEB.):147-53.
65. Tarsitani L, Pasquini M, Maraone A, Zerella MP, Berardelli I, Giordani R, et al. Acute psychiatric treatment and the use of physical restraint in first-generation immigrants in Italy: a prospective concurrent study. Int J Soc Psychiatry. 2013;59(6):613-8.
66. Thomas CS, Stone K, Osborn M, Thomas PF, Fisher M. Psychiatric morbidity and compulsory admission among UK-born Europeans, Afro-Caribbeans and Asians in Central Manchester. Br J Psychiatry. 1993;163(7):91-9.
67. Thornicroft G, Davies S, Leese M. Health service research and forensic psychiatry: A black and white case. Int Rev Psychiatry. 1999;11(2-3):250-7.
68. Tolmac J, Hodes M. Ethnic variation among adolescent psychiatric in-patients with psychotic disorders. Br J Psychiatry. 2004;184(MAY):428-31.
69. Webber M, Huxley P. Social exclusion and risk of emergency compulsory admission. A case-control study. Social Psychiatry and Psychiatric Epidemiology. 2004;39(12):1000-9.
70. Weich S, McBride O, Twigg L, Duncan C, Keown P, Crepaz-Keay D, et al. Variation in compulsory psychiatric inpatient admission in England: a cross-classified, multilevel analysis. The Lancet Psychiatry. 2017;4(8):619-26.
71. Wheeler A, Robinson E, Robinson G. Admissions to acute psychiatric inpatient services in Auckland, New Zealand: a demographic and diagnostic review. N Z Med J. 2005;118(1226):U1752.
